# Tightly yet Dynamically Bound Aliphatic Guanidinium Ligands for Lead Halide Perovskite Nanocrystals

**DOI:** 10.1021/jacs.5c09354

**Published:** 2025-09-17

**Authors:** Yuliia Berezovska, Sebastian Sabisch, Caterina Bernasconi, Yesim Sahin, Federica Bertolotti, Antonietta Guagliardi, Maryna I. Bodnarchuk, Dmitry N. Dirin, Maksym V. Kovalenko

**Affiliations:** † Institute of Inorganic Chemistry, Department of Chemistry and Applied Biosciences, 27219 ETH Zürich, CH-8093 Zürich, Switzerland; ‡ Empa−Swiss Federal Laboratories for Materials Science and Technology, CH-8600 Dübendorf, Switzerland; § Dipartimento di Scienza e Alta Tecnologia & To.Sca.Lab, Università dell’Insubria, 22100 Como, Italy; ∥ Istituto di Cristallografia & To.Sca.Lab, Consiglio Nazionale delle Ricerche, 22100 Como, Italy

## Abstract

Lead halide perovskites (LHPs) have disrupted the field of visible-range emitting colloidal semiconductor nanocrystals (NCs) owing to their exceptional emissivity as classical and quantum light sources. Their appeal is countered by the stability challenges arising from inherent lattice softness and labile surface ligand bonding. Recent ligand design efforts have drastically improved the structural integrity of LHP NCs by pursuing strongly and statically bound ligands, as found with zwitterionic headgroups. Here, we venture into a new class of cationic ligands featuring guanidinium headgroups, which combine the compactness of primary ammoniums with the deprotonation resistance of quaternary ammoniums. We reasoned that strong yet dynamic ligand binding offers distinct benefits. A library of aliphatic guanidinium molecules was synthesized and surveyed as capping ligands for CsPbBr_3_, FAPbBr_3_, and CsPbI_3_ NCs, enabling up to 95% photoluminescence quantum yields for bromide compositions. We then compare binding behavior of guanidinium ligands with those of other widely used ligands. Zwitterionic ligands phosphocholines and phosphoethanolamines bind more strongly than sulfobetaines and quaternary ammonium ligands, yet all these headgroups impart relatively static binding with exchange rates below 5 s^–1^. Primary ammonium ligands are champions in dynamicity but lag behind in ligand-coverage retention and, hence, long-term stability. Guanidinium ligands strike a favorable balance: they match the binding dynamics of primary ammonium ligands while significantly enhancing binding strength, enabling rigorous purification and compatibility with relatively polar solvents such as tetrahydrofuran. We then showcase the benefits of dynamically bound ligands in photocatalytic C–C coupling reactions.

## Graphical abstract

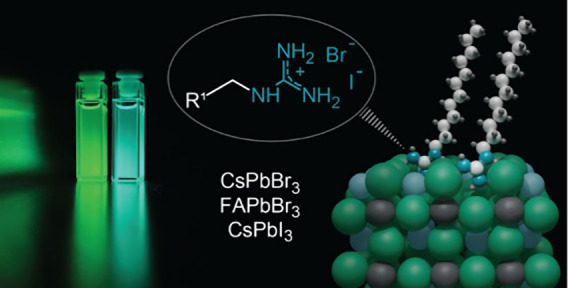

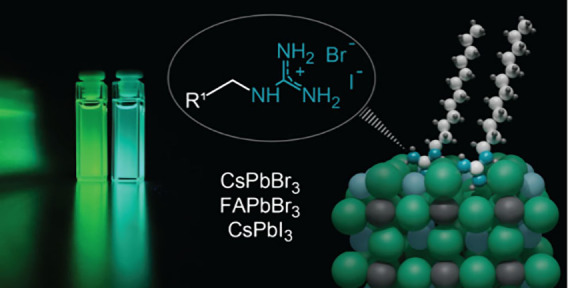

## Introduction

Lead-halide perovskite nanocrystals (LHP NCs) with the composition APbX_3_ (where A = Cs, methylammonium MA, or formamidinium FA; X = Cl, Br, I) have become appealing as classical light sources for backlit and LED displays^1–8^ as well as quantum light sources.^9–14^ The broad utility of LHP NCs arises from a combination of accessible synthetic approaches at both lab and industrial scales,^15–18^ near-unity photoluminescence quantum yields (PLQYs) even at large NC sizes (above Bohr exciton diameter),^19,20^ and facile composition tuning, enabling emission wavelength control across the visible and near-infrared range,^21–31^ while retaining sub-100 meV emission line widths.^32^ Additionally, they offer bright triplet-dominated emission at low temperatures^33^ and ultrafast (sub-100 ps) radiative decay times nearly matching the exciton coherence times (*ca*. 80 ps for large CsPbBr_3_ NCs).^11,34^

Unlike conventional chalcogenide and pnictide semiconductor NCs, the low formation energy of LHPs translates into enhanced structural dynamics and a highly labile surface.^35^ In addition, the electronic structure of LHPs is intrinsically tolerant to A-cation vacancies and strained bonds.^36,37^ This interplay between structural dynamics and defect tolerance enables LHP NCs to accommodate substantial surface distortions and exhibit dynamic ligand binding without notably compromising optical properties,^23,38,39^ thereby facilitating the development of diverse surface chemistries and a broad range of functional ligands.^19,40–52^ At the same time, the low lattice energy of LHPs sets an upper limit on the bond strength between the NC surface and ligand headgroups,^35^ resulting in comparatively weak and often dynamic ligand binding.^38,46^

The first—and now canonical though suboptimal—ligand applied to LHP NCs is oleylammonium (OAmH^+^), which replaces surface A-site cations and primarily interacts ionically with the surface, often alongside carboxylates (*e.g.*, oleate, OAc^–^).^39,45–47,53^ The labile surface chemistry and ionic binding of alkylammonium ligands enable their dynamic desorption via deprotonation (OAmH^+^ + OAc^–^ ⇋ OAm + OAcH) or salt formation (OAmH^+^ + OAc^–^ ⇋ OAmHOAc or OAmH^+^ + Br^–^ ⇋ OAmHBr).^38^ Early generations of LHP NCs, capped with a combination of oleylamine and oleic acid (OAm/OAcH), were particularly susceptible to deprotonation-induced instability, leading to a loss of PLQY, as well as colloidal and structural integrity when exposed to polar solvents during purification.^19,39^ Fully alkyl-substituted ammonium, phosphonium, and sulfonium ligands offer enhanced robustness due to resistance to deprotonation.^19,54–56^ While purely cationic ligands offer distinct advantages, such as selective binding to A-sites without contributing to the density of states near the band edges, further improvements in stability required leveraging the entropy-driven chelate effect.^40,45^ In this vein, zwitterionic ligands based on sulfobetaine, γ-amino acid, phosphocholine (PC), and phosphoethanolamine (PEA) headgroups reinforce binding through the chelate effect, enhancing compatibility with mildly polar solvents and enabling more rigorous purification.^40,43,45,57^

Ligands with diverse headgroups exhibit a range of binding dynamics and strengths at LHP NC surfaces.^43,58,59^ While binding strength governs the equilibrium between bound and free ligands in solution, the rates of ligand adsorption and desorption are determined by the activation barriers associated with the transition state. Consequently, ligands can exhibit one of four binding modes: strong-static, strong-dynamic, weak-static, or weak-dynamic, with the first two being of practical use. Although most research efforts focused on ligands that bind strongly and statically, we recognize that dynamic ligand binding can enhance surface accessibility—an essential factor in photoredox catalysis—and can therefore be advantageous, provided stability is maintained.

Pursuing dynamically yet tightly binding ligands, we revisit purely cationic molecules and propose guanidinium (GA) as a new headgroup. GA combines key advantages of both primary and quaternary ammonium headgroups. Similar to primary ammonium, GA fits well into the A-site pocket on the NC surface; yet, like a quaternary ammonium, it resists deprotonation. As a result, GA-based ligands combine dynamic surface binding with enhanced stability against polar solvents. A library of GA-based ligands with varied tail functionalities was developed and successfully applied to LHP NCs of different sizes and compositions, achieving colloidal stability even in moderately polar solvents such as tetrahydrofuran (THF). We then systematically compare the binding dynamics and strength of guanidinium and other ligands: primary and quaternary ammonium salts, as well as zwitterionic ligands based on sulfobetaine, phosphocholine, and phosphoethanolamine. Specifically, we analyzed ligand diffusion in native colloidal solutions using diffusion-ordered nuclear magnetic resonance spectroscopy (DOSY NMR) of CsPbBr_3_ NCs with varied ligand concentrations. We find that the ligand headgroup dictates binding dynamics, with GA-ligands offering a balance between high dynamicity and excellent NC stability. Finally, we demonstrate the potential of dynamically bound ligands in photocatalytic applications, exemplified by C–C homocoupling reaction, where GA-based ligands significantly outperform more static ligands.

## Results and Discussion

In a search for alternative cationic ligands for LHP NCs, we hypothesized that guanidinium (GA) could serve as an effective headgroup combining the advantages of quaternary and primary ammonium fragments. GA is resistant to deprotonation due to its high p*K*_a_ (13.6 vs. 9–10 for primary ammonium),^60^ provides multiple hydrogen bonding sites to coordinate with surrounding halide anions,^61,62^ and possesses a comparatively small ionic radius (278 pm vs. 292 pm for quaternary ammonium).^63^ While the GA cation itself is still too large to fit into the 3D LHP lattice,^62^ the surface footprint of its monoalkylguanidinium derivative can be accommodated. Geometry-optimized DFT models of CsPbBr_3_ NCs capped with a single primary ammonium, GA, or quaternary ammonium ligands with their respective tails (two C_12_ tails for the quaternary ammonium and one oleyl C_18_ for both the primary ammonium and GA), show that the GA headgroup is a better geometrical fit to the NC surface than the quaternary ammonium group (Figure 1a). The improved surface fit can be demonstrated by the shorter optimal distance between the central atom of the ligand headgroup and the nearest Cs^+^ cation beneath the surface site (6.6 Å for GA vs 7.5 Å for quaternary ammonium).^45^

**Figure 1:**
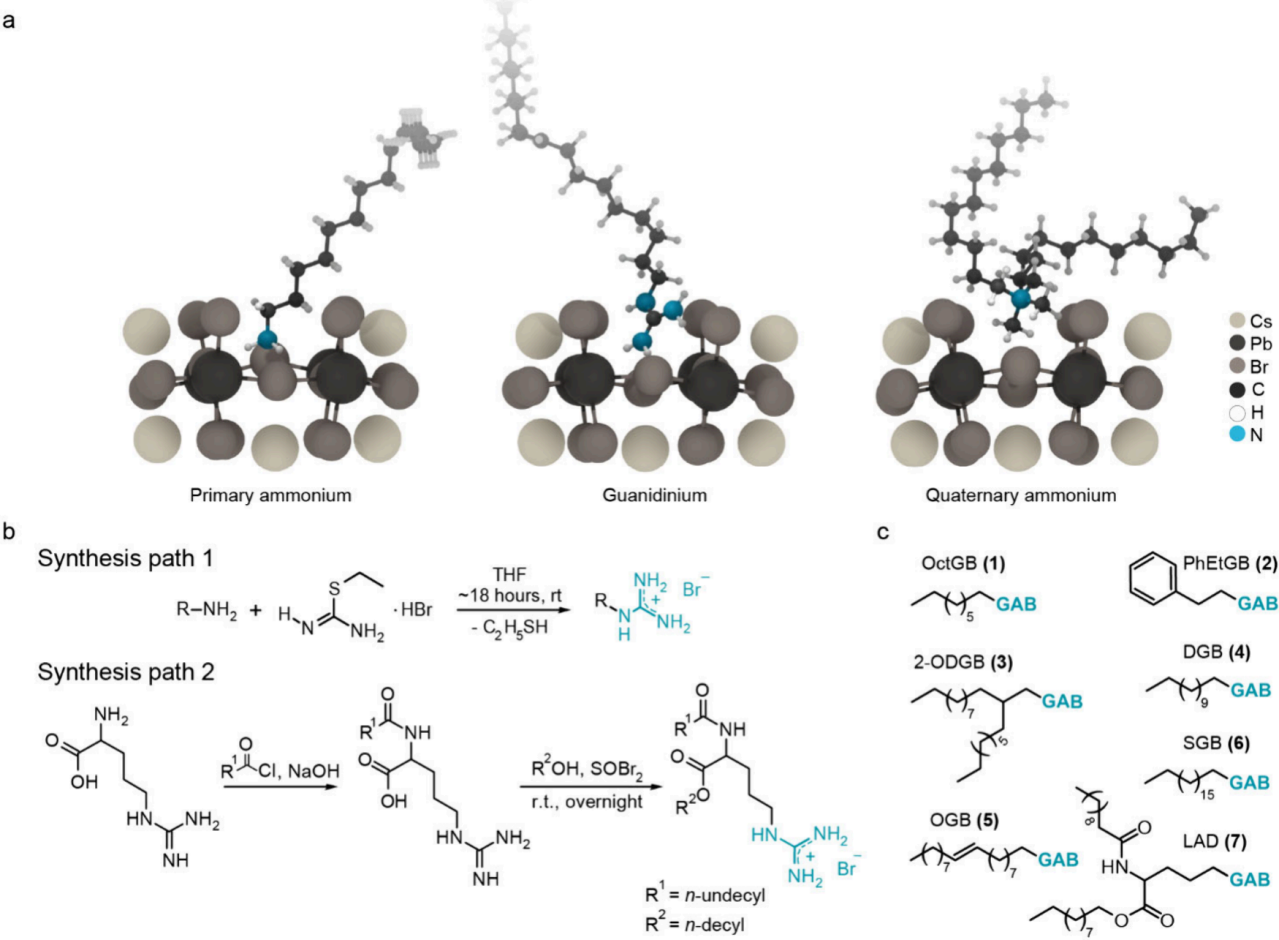
(a) DFT models of ligand surface configurations with primary ammonium, guanidinium, and quaternary ammonium illustrating the perfect surface fit of the first two. (b) Synthesis scheme for the guanidium-based ligands via guanidinylation of primary amines (path 1, R corresponds to the tails 1–6 depicted in (c)) or functionalization of arginine (path 2). (c) Synthesized ligands with guanidinium bromide headgroup (GAB): *N*-octyl guanidinium bromide (1, OctGB), phenethyl guanidinium bromide (2, PhEtGB), *N*-(2-octyl dodecyl) guanidinium bromide (3, 2-ODGB), *N*-dodecyl guanidinium bromide (4, DGB), *N*-(octadec-9-en-1-yl) guanidinium bromide (5, OGB), *N*-octadecyl guanidinium bromide (6, SGB), and lauroyl-arginine-decane (7, LAD).

We developed a versatile synthetic approach to access a diverse library of GA-based ligands with tailored tail functionalities. Most ligands were synthesized via a one-step reaction between isothiouronium salts and various aliphatic amines (Figure 1b, Path 1).^64–66^ This procedure enables the attachment of diverse tail groups to the GA group and allows customization of the halide counterion. An alternative two-step synthesis, starting from arginine, was employed for ligands with additional branching at the fourth carbon (Figure 1b, Path 2). Using these methods, we synthesized seven ligands with varied functionalities and degrees of branching, each prepared directly as a halide salt (Figure 1c). These include *N*-octyl guanidinium bromide (**1**, OctGB), phenethyl guanidinium bromide (**2**, PhEtGB), *N*-(2-octyl dodecyl) guanidinium bromide (**3**, 2-ODGB), *N*-dodecyl guanidinium bromide (**4**, DGB), *N*-(octadec-9-en-1-yl) guanidinium bromide (**5**, OGB), *N*-octadecyl guanidinium bromide (**6**, SGB), and lauroyl-arginine-decane (**7**, LAD). The majority of these salts feature aliphatic tail functionalities with PhEtGB (**2**) included for comparison.

GA-based ligands were first employed in the hot-injection synthesis of colloidal CsPbBr_3_ NCs, serving both as surface ligands and as a bromide source. Lead and cesium salts were dissolved in mesitylene with an excess of hexanoic acid at 90 °C under a nitrogen atmosphere, followed by injection of a mesitylene solution containing the GA-based molecules (Figure 2a). The hot-injection method yielded CsPbBr_3_ NCs with an average size of approximately 9 nm, a narrow size distribution, and PLQYs of up to 95% (Figures 2b, 2c). The short-chain ligands OctGB (**1**) and PhEtGB (**2**) yielded bright, colloidally stable crude NC solutions but did not impart sufficient steric repulsion to allow for successful purification. In contrast, SGB (**6**) led to aggregation already in the crude solution, likely due to ligand crystallization on the NC surface. All other GA ligands produced colloidally stable crude solutions and permitted purification, resulting in colloidally stable solutions in cyclohexane and THF. Among these, OGB (**5**) and LAD (**7**) yield the highest PLQY and best colloidal stability (Figures 2b, 2c). Cyclohexane solutions of these NCs endure at least three purification cycles with methyl acetate, exhibiting only a mild reduction in PLQY, and remain stable for over six months under ambient conditions. Both ligands allow NC dispersions with high concentrations (>80 mg/mL) and maintain a striking PLQY in NC films (75% for OGB (**5**) and 85% for LAD (**7**)).

**Figure 2:**
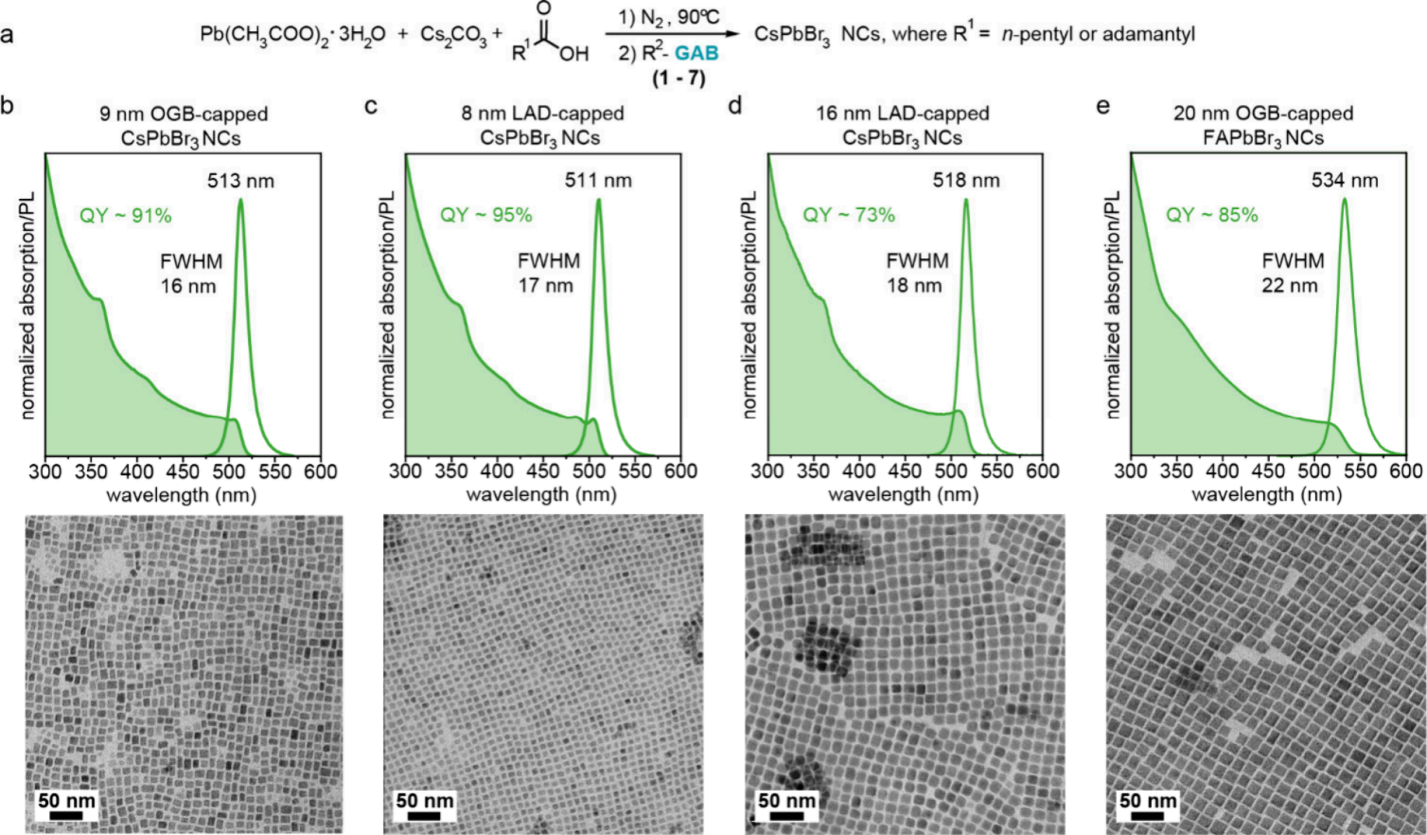
(a) Reaction scheme for the hot-injection synthesis of CsPbBr_3_ NCs, employing guanidinium-based molecules as both ligands and a bromide source. (b-e) Representative normalized absorption and photoluminescence (PL) spectra of 9 nm large OGB-capped CsPbBr_3_ NCs, 8 nm large LAD-capped CsPbBr_3_ NCs, 16 nm large LAD-capped CsPbBr_3_ NCs, and 20 nm large OGB-capped FAPbBr_3_ with corresponding TEM images (bottom panel).

We found that the bulkiness of the ligands has minimal influence on NC nucleation and growth, with the shorter DGB (**4**), longer OGB (**5**), and branched 2-ODGB (**3**) and LAD (**7**) ligands yielding NCs ranging from 8 to 11 nm in size (Figures 2b, 2c, and S1). Annealing LAD-capped NCs in the crude solution yields larger truncated cubes (∼16 nm) with a PLQY of 73% (Figure 2d). Alternatively, replacing hexanoic acid with oleic acid introduces a moderately effective ligand that reduces NC size to below 5 nm (Figure S2), promoting a slightly prolate morphology with an aspect ratio of up to 1.7, as compared to an aspect ratio of 1.2–1.3 observed for larger NCs (Figure S3). This shape transformation is supported by transmission electron microscopy (TEM) and total X-ray scattering analysis (Tables S1, S2, and Figure S4).^67^

GA-based ligands enable compositional tunability at both the A- and X-sites. Colloidally stable ∼20 nm OGB-capped FAPbBr_3_ NCs with a PLQY of ∼85% were obtained (Figure 2e), although hexanoic acid had to be replaced with bulky adamantanoic acid to account for the generally faster growth kinetics of LHP NCs with organic A-site cations and to prevent the formation of bulk FAPbBr_3_. Similarly, ∼20 nm CsPbI_3_ NCs with narrow size distribution were synthesized using the corresponding iodide salt, oleylguanidinium iodide (OGI, see SI for the details on synthesis). Importantly, these NCs can be purified under ambient conditions and retain their integrity for months (Figure S5). We note that, to our knowledge, only the primary-ammonium-assisted synthesis had thus far yielded purifiable, phase-stable perovskite FAPbI_3_ and CsPbI_3_ NCs in their luminescent phase.^29,40,68–70^

NCs obtained via hot-injection synthesis are capped exclusively with GA-based ligands, as confirmed by solution NMR spectroscopy experiments. After the first purification cycle, hexanoic acid is removed to undetectable levels, as evidenced by the absence of the characteristic peak at ∼12.25 ppm in the NMR spectrum (Figure S6). By the third purification cycle, the reaction solvent is also eliminated, leaving signals corresponding solely to OGB (**5**) and the purification solvent. Digestion of the NCs with DMSO-*d*_6_ further confirms the absence of any coordinating species other than OGB (**5**), verifying it as the sole organic molecule present on the CsPbBr_3_ NC surface (Figure S7). Additionally, the ^1^H NMR spectrum of purified OGB-capped NCs (Figure S6) does not clearly display signals corresponding to the terminal NH proton (7.0–7.75 ppm) or the backbone NH proton (7.8 ppm), as these are significantly broadened due to a combination of chemical exchange and the reduced tumbling rate of the NCs, making them difficult to detect.

Building on the excellent colloidal stability and optical performance enabled by GA-based ligands in hot-injection synthesis, we extended their application to a recently developed room-temperature synthesis of LHP NCs that offers improved control over NC size and uniformity.^15^ Initially, CsPbBr_3_ NCs are grown in the presence of weakly binding ligands, such as tri*-n*-octylphosphine oxide and diisooctylphosphinate, allowing for slow and controlled crystal growth (Figure 3a). Once the desired NC size is reached, a stronger GA ligand is introduced for stabilization and purification. This approach enabled the synthesis of monodisperse OGB- or LAD-capped CsPbBr_3_ NCs with tunable sizes ranging from 5 to 11 nm (Figures 3b, 3e). As in the hot-injection synthesis, GA headgroup promotes the growth of cuboidal NCs despite the initial pseudospherical morphology typical for this synthetic method.^15^ These NCs were purified using methyl acetate and redissolved in cyclohexane or THF. Shorter OctGB (**1**) and PhEtGB (**2**) failed to provide the colloidal stability needed for purifying NCs from the crude solution. However, their ability to quench NC growth suggests that they still bind to NC surfaces. Notably, until recently, growth quenching and stabilization in this room-temperature synthesis relied exclusively on zwitterionic ligands.^71^

**Figure 3:**
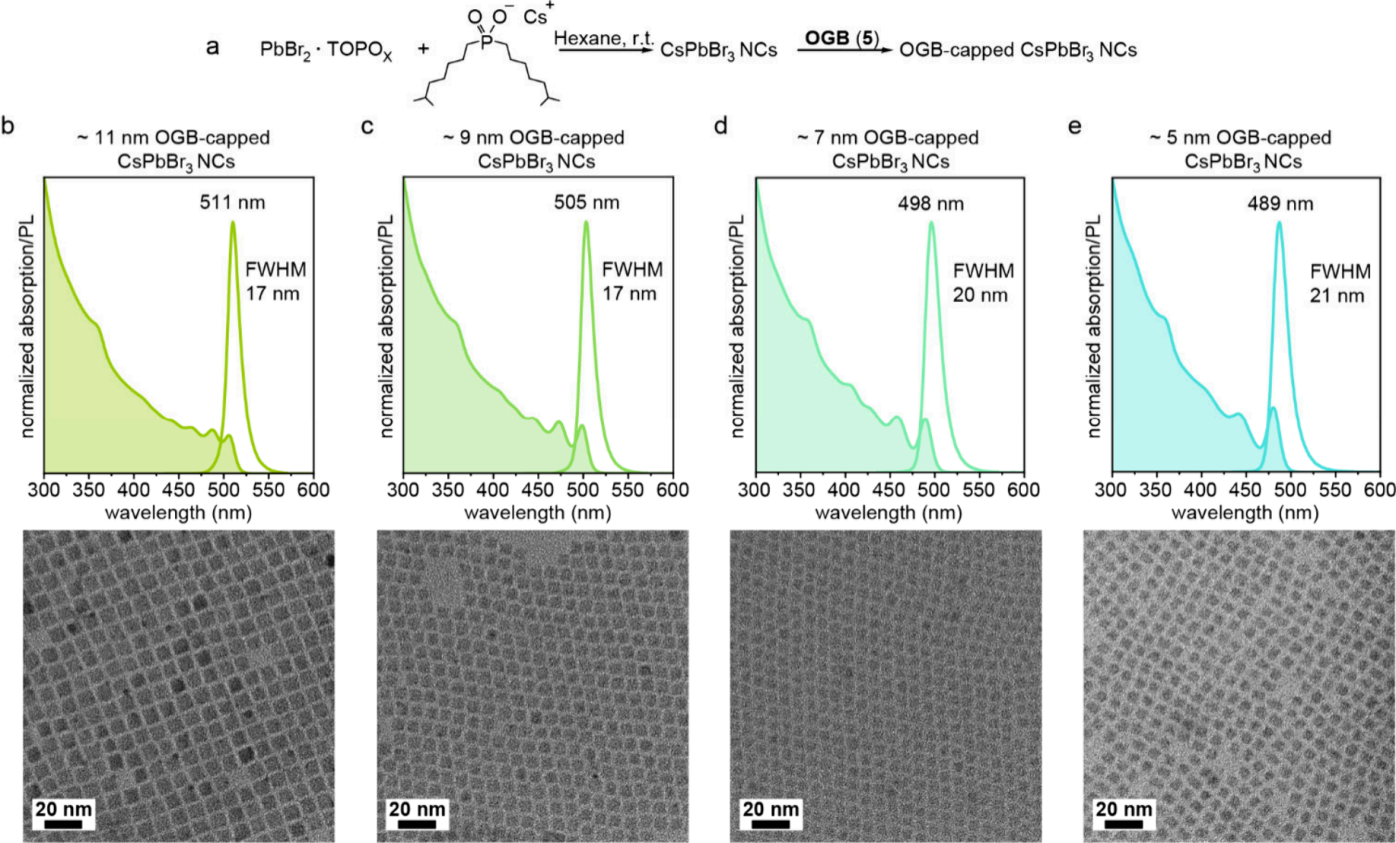
(a) Reaction scheme for the room-temperature synthesis of CsPbBr_3_ NCs. (b-e) Representative normalized absorption and PL spectra of OGB-capped 11, 9, 7, and 5 nm large CsPbBr_3_ NCs with corresponding TEM images (bottom panel).

We then surveyed the binding dynamics of the developed GA-based ligands alongside other ligand classes commonly used for LHP NCs. For that, we employed DOSY NMR, which differentiates overlapping NMR signals of free and NC-bound ligands based on their diffusion coefficients.^72^ Free ligands exhibit diffusion coefficients of approximately 10^–9^ m^2^/s, while statically bound ligands—associated with the significantly larger solvodynamic radii of the colloid over the free ligand—show lower diffusion coefficients (∼10^–11^ m^2^/s, depending on NC size) (Figure 4a). In NC solutions, ligands continuously adsorb to and desorb from the NC surface, with exchange rates governed by the respective activation barriers (Figure 4b). DOSY NMR can distinguish two scenarios that depend on the kinetics of this exchange. When ligands are in a rapid adsorption/desorption equilibrium, *i.e.* dynamically bound, the time scale of ligand exchange may be shorter than that of the measurement. Consequently, the measurement captures an effective diffusion coefficient reflecting the weighted contributions of both free and bound ligand populations, characterized here by the fraction of bound ligands *f* (Figure 4c, left). In contrast, when ligands are more statically bound, signal attenuation exhibits two distinct components corresponding to free (fast-diffusing) and bound (slow-diffusing) ligands in solution (Figure 4c, right). For these systems, the ratio of bound to free ligands corresponds to the ratio of the signal attenuation components rather than to changes in the diffusion coefficient.

**Figure 4:**
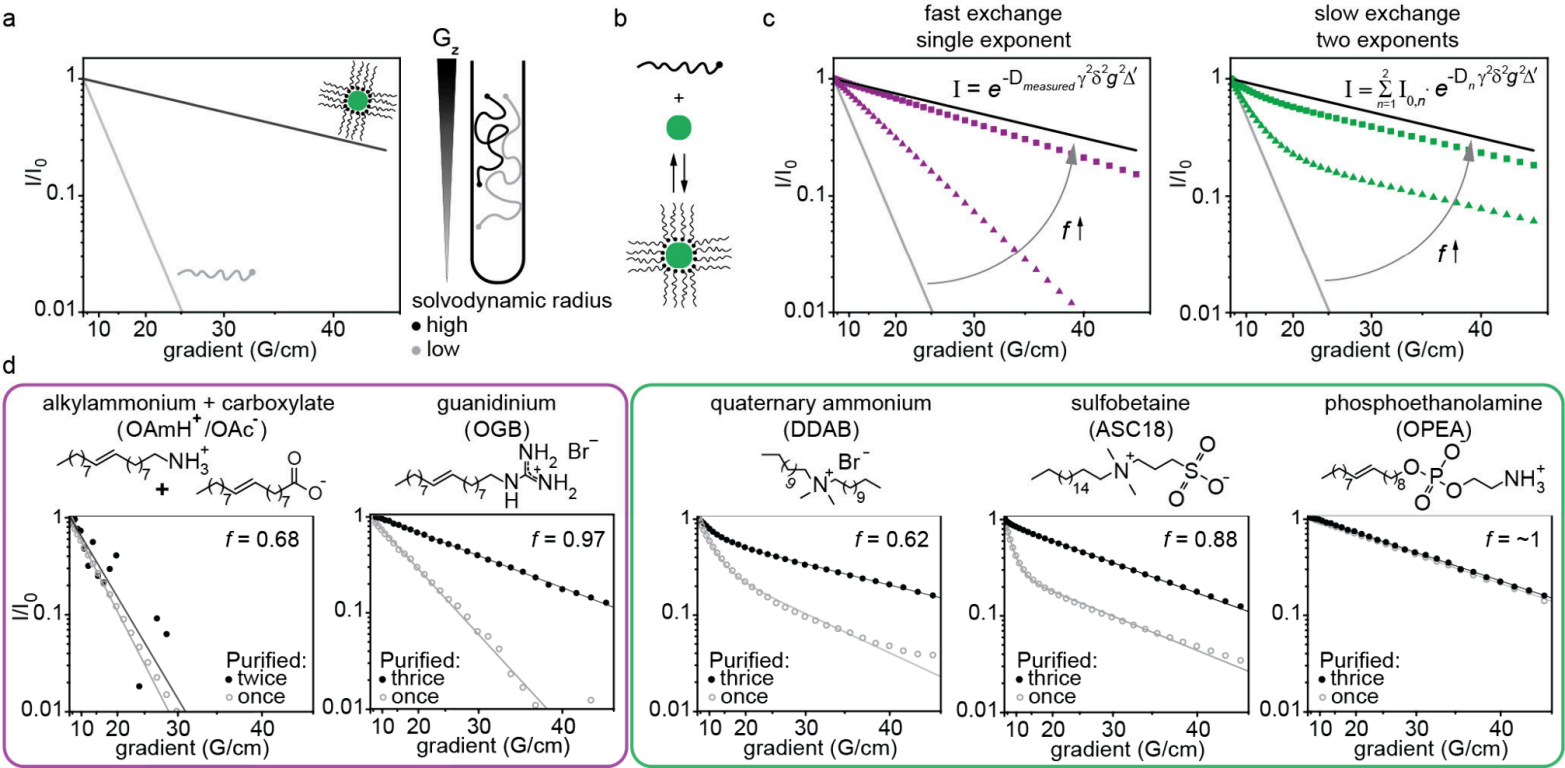
(a) The modeled DOSY NMR curve for free ligands with a small solvodynamic radius follows a fast single-exponential decay (gray curve). In contrast, ligands that are statically bound to the NC surface exhibit a slow single-exponential decay (black curve) due to their larger effective solvodynamic radius. (b) Schematic of the dynamic equilibrium between free and bound ligands. (c) Modeled DOSY NMR curves for weakly and strongly bound ligands undergoing fast exchange (purple), and similarly weakly and strongly bound ligands undergoing exchange slower than the DOSY NMR time scale (green). Equations illustrate mono- and bicomponent attenuations, respectively: *D* - diffusion coefficient, γ - gyromagnetic ratio, δ - length of the gradient pulses (δ = 4 ms), *g* - gradient strength, and Δ - diffusion time. (d) Experimental DOSY NMR curves for the ligand families featuring different headgroups: oleylammonium and oleate (OAmH^+^/OAc^–^), oleylguanidinium (OGB), dimethyldidodecylammonium bromide (DDAB), 3-(*N, N*-dimethyloctadecylammonio) propanesulfonate (an ammonio sulfonate with a C18 alkyl tail, herein denoted as ASC18), and 2-ammonioethyl oleyl phosphate (or oleylphosphoethanolamine, OPEA). DOSY NMR curves are recorded after one or several purification steps to illustrate the difference in binding dynamics at varying ligand concentrations. The fitting procedure can be found in the SI.

As a result, for thoroughly purified NCs with ligands in binding equilibrium and incomplete surface coverage, DOSY NMR provides two distinct insights. (1) It quantifies the fraction of bound ligands *f*, which reflects the desorption equilibrium constant and, via the Gibbs free energy of desorption, the ligand binding strength—allowing ligands to be ranked accordingly. (2) It probes ligand binding dynamicity by comparing exchange rates to the DOSY NMR time scale, as revealed by the form of signal attenuation (mono- vs. biexponential). The experiment time scale is defined by the diffusion time.^73^ The diffusion time was screened between 200 and 700 ms (see Figure S8 and corresponding discussion in the SI for details) and fixed at 200 ms, enabling discrimination between ligands undergoing exchange faster or slower than approximately 5 s^–1^

DOSY NMR spectra of CsPbBr_3_ NCs capped with different ligands were recorded immediately after the first purification step (precipitation with antisolvent from the crude solution) and again after multiple purification cycles. Purification facilitates the removal of free ligands, thereby altering the ratio of bound to free ligands. Commonly used purification protocols were applied for each ligand, involving three cycles whenever possible without compromising NC integrity. All NCs were dissolved in benzene-d_6_ except for the samples capped with didodecyldimethylammonium bromide (DDAB) and 2-ammonioethyl 2-octyl-1-dodecyl phosphate (ligand with a branched tail and phosphoethanolamine headgroup, brPEA), which were dissolved in cyclohexane-d_12_ due to poor solubility in benzene. Detailed protocols and fits are available in the Supporting Information.

As expected, NCs capped with the OAmH^+^/OAc^–^ ligand pair exhibited the most dynamic ligand binding. After the first purification cycle, the remaining ligands in the solution exhibit fast single-component diffusion comparable to that of free ligands (Figure 4d), indicating a rapid exchange between bound and free states. The equilibrium was slightly shifted toward the bound state (*f* = 0.64), consistent with previous reports.^38^ Further purification did not significantly alter the effective diffusion coefficient, but instead compromised NC stability, suggesting that additional purification primarily removes surface-bound ligands without shifting the equilibrium (Figure 4d, *f* = 0.68). Such behavior aligns with the rapid loss of PLQY and eventual NC disintegration observed under thorough purification.

In contrast, NCs capped with OGB ligands (**5**) tolerated three purification cycles while exhibiting a notable reduction in the free ligand fraction (Figure 4d). The spectra of once-precipitated NC solutions revealed a single component indicative of fast dynamic binding (*D*_OGB_ = 1.4 × 10^–10^ m^2^/s). Subsequent purification shifted the equilibrium further toward bound ligands (*f* reaching 0.97), resulting in slower yet still single-component diffusion attenuation for OGB-capped NCs (*D*_OGB_ = 5.0 × 10^–11^ m^2^/s; Figure 4d).

NCs capped with quaternary ammonium ligands DDAB also tolerated three purification cycles while exhibiting a notable reduction in the free ligand fraction (*f* increasing from 0.38 to 0.67). However, unlike OGB, they displayed two-component signal attenuation, revealing slower binding dynamics compared to the OAmH^+^/OAc^–^ ligand pair and OGB. A qualitatively similar behavior was observed for NCs capped with sulfobetaine ligands (3-(*N, N*-dimethyloctadecylammonio) propanesulfonate, an ammonio sulfonate with a C18 alkyl tail, herein denoted as ASC18), although with a higher fraction of bound ligands *f* = 0.88 after three purification cycles, indicating stronger binding of ASC18 compared to DDAB. The combination of a high bound-ligand fraction and slow exchange dynamics corroborates the enhanced stability of ASC18-capped NCs.

For NCs capped with 2-ammonioethyl oleyl phosphate—a ligand featuring an oleyl tail and phosphoethanolamine headgroup (OPEA)—the first precipitation from the crude solution effectively removed most of the free ligands (Figure 4d). As a result, ligands in these solutions exhibited exclusively particle-like diffusion coefficients, comparable to the slow component observed for DDAB- and ASC18-capped NCs. Further purification did not alter the diffusion coefficients or compromise the colloidal stability of NCs, indicating minimal detachment of the bound ligands. The near-unity fraction of bound phospholipid ligands obscures their presumably static binding, as the free ligand signal, corresponding to the fast component in diffusion attenuation, remained below the detection limit of DOSY NMR. To confirm that phospholipid ligands bind statically to the NC surface, we deliberately added free ligands to the solution and, as expected, observed two-component diffusion attenuation (Figure S10). These findings align with the reported enhanced stability of NCs capped with zwitterionic ligands, particularly those based on phospholipids.^45^

Analogous diffusion behavior was observed for other tested phospholipid ligands, including (2-(trimethylammonio)­ethyl) oleyl phosphate (a ligand with an oleyl tail and phosphocholine headgroup, OPC), brPEA, and lecithin (Figures S11–13). For OPC and brPEA, this behavior indicates similarly strong and static binding. In the case of lecithin—the most commonly used PC-based ligand for LHP NCs—we found that unbound ligand molecules form micelles in benzene even at concentrations as low as 2.2 mM (Figure S13c). As a result, the diffusion coefficient of nominally free lecithin is low and comparable to that of bound ligand, preventing a definitive determination of its binding character. Nevertheless, given the extensive experimental evidence supporting the high colloidal stability of lecithin-capped NCs,^43,58,59^ and the general observation that binding behavior is primarily dictated by the headgroup, we conclude that lecithin binds strongly and statically to the CsPbBr_3_ NC surface, consistent with other phospholipid ligands.

To elucidate the role of the GA headgroup in the dynamic binding and decouple it from possible effects of the ligand tail, OGB (**5**) was compared with OPEA—a ligand featuring the same oleate tail but phospholipid headgroup, which has demonstrated more static binding in DOSY NMR (Figure 4d). Unlike OGB (**5**), OPEA-capped NC solutions exhibited a minimum amount of free ligands already after the first purification cycle and maintained a stable concentration of both ligands and NCs across additional purifications (Figure S14a). OGB-capped NCs, on the other hand, exhibited a decline in ligand concentration upon purification, as free ligands were removed with the supernatant (Figure S14b). This contrast highlights the dominant role of the ligand headgroup in ligand binding dynamicity. Interestingly, we found that the introduction of amide and ester fragments into the organic tail could also slightly alter ligand binding behavior, likely due to enhanced hydrogen bonding between the ligands or interaction of the ester groups with the undercoordinated subsurface Pb atoms.^74^ As a result, LAD-capped NCs (**7**), despite incorporating GA headgroup, exhibited two distinct diffusion coefficients regardless of the number of purification cycles (*D*_LAD‑Fast_ = 5.0 × 10^–10^ m^2^/s and *D*_LAD‑Slow_ = 4.7 × 10^–11^ m^2^/s and *f* = 0.58; Figure S15).

Based on our findings, we classify ligand families into two groups according to their binding dynamicity and strength:

Desorption rate faster than 5 s^–1^ (dynamically bound ligands):
  primary ammonium ≪ guanidinium
Desorption rate slower than 5 s^–1^ (less dynamically bound ligands):
  quaternary ammonium < C_3_-sulfobetaine ≪C_2_-phosphocholine ∼ C_2_-phosphoethanolamine

The observed free/bound ligand exchange rates are in agreement with the rates of monomer incorporation into phospholipid bilayer, typically ranging from 10^–6^ to 10^–1^ s^–1^ at room-temperature,^75–77^ and previously reported rates of ligand-mediated cross-anion exchange between CsPbBr_3_ and CsPbCl_3_ NCs: 3·10^–3^ s^–1^ for lecithin and 16·10^–3^ s^–1^ for ASC18.^58^

The exceptional combination of dynamic surface binding and NC resilience, even at low surface coverage, makes GA-based ligands particularly appealing for photocatalytic applications, where both surface accessibility and stability are essential for efficient catalytic transformations.^78^ The impact of ligand binding dynamics on photocatalytic performance was evaluated by directly comparing OGB- and OPEA-capped NCs. For completeness, ASC18—a ligand with the same tail length as OGB (**5**) and OPEA but featuring a sulfobetaine headgroup—was also included to cover a broader range of binding behaviors. Photocatalytic performance was assessed using the reductive C–C bond coupling of benzyl bromide, a reaction compatible with solvents of varying polarity, from cyclohexane to *n*-butanol.^20,79^ To ensure a fair comparison, experiments were conducted in benzene, which supports colloidal stability for NCs capped with all three ligands (Figure S16). The photocatalyst loading was kept low to avoid complete conversion, isolating the influence of ligand type on performance.

As anticipated, OGB-capped NCs exhibited twice the catalytic efficiency of OPEA-capped NCs (Figure 5). This comparison of ligands featuring the same tail, but different headgroups and binding behavior represents the first direct demonstration of how ligand binding dynamics influence the photocatalytic performance of colloidal NCs. ASC18-capped NCs, while less efficient than OGB-capped ones, still outperformed NCs capped with more strongly bound OPEA ligands in benzene.

**Figure 5:**
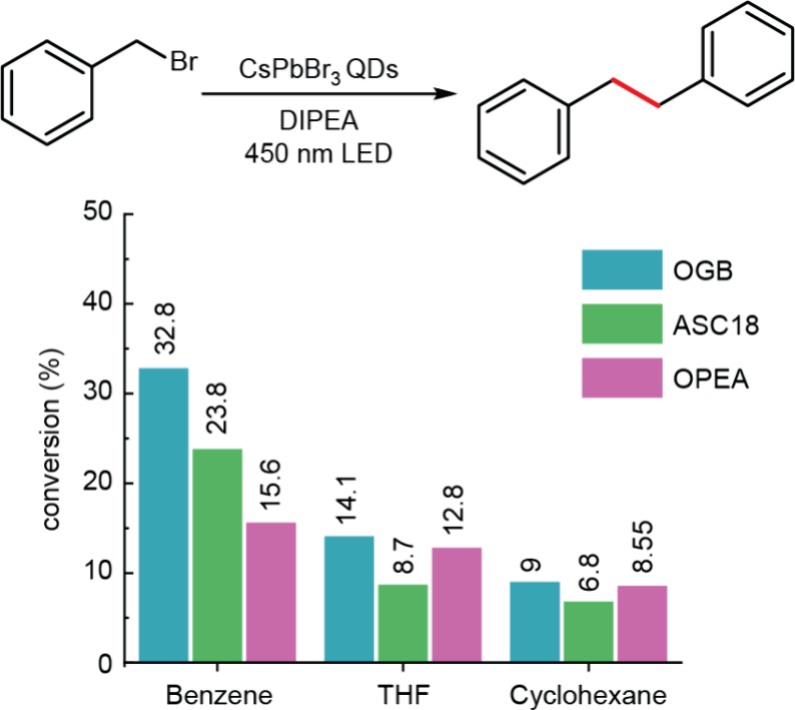
Scheme of photocatalytic reaction and corresponding conversion to product for OGB-capped CsPbBr_3_ NCs (teal), ASC18-capped CsPbBr_3_ NCs (green), and OPEA-capped CsPbBr_3_ NCs (pink) in various solvents (benzene, THF, cyclohexane).

In other solvents (THF and cyclohexane), CsPbBr_3_ NCs capped with OGB (**5**), ASC18, and OPEA show more complex trends in conversion efficiency, which also reflect differences in the colloidal stability of NCs enabled by the respective ligands. However, even in these cases, OGB-capped NCs exhibit the highest conversion.

These results align with recent studies reporting superior performance of dynamically bound ASC18 in photocatalytic C–H brominations of alkylarenes, ketones, and β-ketoesters.^78^ Collectively, these findings confirm that the enhanced performance of NCs capped with dynamically bound ligands, such as OGB (**5**) and ASC18, is not reaction-specific, provided NC stability is maintained. They also underscore the potential for tailoring GA-based ligands to further optimize photocatalytic processes.

## Conclusion

We introduce aliphatic guanidinium salts as a new class of ligands for LHP NCs, enabling strong yet dynamic surface binding. Versatile synthetic strategies were developed to construct a library of guanidinium-based ligands with diverse tail functionalities. These ligands enable the synthesis of CsPbBr_3_, FAPbBr_3_, and CsPbI_3_ NCs with tunable sizes ranging from 5 to 20 nm and PLQY reaching up to 95% in solution and 80% in films for bromide-based NCs. Using DOSY NMR, we systematically compared the binding strength and dynamics of commonly used ligands alongside the new guanidinium-based molecules. We confirm that binding behavior is governed primarily by the ligand headgroup, with behavior ranging from highly dynamic, weakly bound primary ammonium ligands to relatively static, strongly bound phospholipids. The guanidinium headgroup offers dynamic—comparable to primary ammonium ligands—yet strong binding and robust colloidal stability, on par with zwitterionic sulfobetaines. This enables NC stability even at low surface coverage and in moderately polar solvents such as THF. The combination of dynamic binding behavior and superior stability makes guanidinium-based ligands particularly well-suited for photocatalytic applications, where high surface accessibility is essential. In model C–C coupling reactions photocatalyzed by CsPbBr_3_ NCs, dynamically yet tightly bound guanidinium-capped NCs exhibited double the catalytic efficiency of their more statically bound counterparts, underscoring their potential for demanding catalytic transformations.

## Supplementary Material



## Notes

Materials, synthesis details, experimental methods, and supplementary figures (PDF)

## References

[1] Akkerman Q. A., Rainò G., Kovalenko M. V., Manna L. (2018). Genesis, challenges
and opportunities for colloidal lead halide perovskite nanocrystals. Nat. Mater..

[2] Shamsi J., Urban A. S., Imran M., De Trizio L., Manna L. (2019). Metal Halide Perovskite Nanocrystals: Synthesis, Post-Synthesis Modifications,
and Their Optical Properties. Chem. Rev..

[3] Liu X.-K., Xu W., Bai S., Jin Y., Wang J., Friend R. H., Gao F. (2021). Metal halide
perovskites for light-emitting diodes. Nat.
Mater..

[4] Veldhuis S. A., Boix P. P., Yantara N., Li M., Sum T. C., Mathews N., Mhaisalkar S. G. (2016). Perovskite
Materials for Light-Emitting
Diodes and Lasers. Adv. Mater..

[5] Wang X., Bao Z., Chang Y.-C., Liu R.-S. (2020). Perovskite Quantum Dots for Application
in High Color Gamut Backlighting Display of Light-Emitting Diodes. ACS Energy Lett..

[6] Dey A., Ye J., De A., Debroye E., Ha S. K., Bladt E., Kshirsagar A. S., Wang Z., Yin J., Wang Y. (2021). State
of the Art and Prospects for Halide Perovskite Nanocrystals. ACS Nano.

[7] Jiang Y., Sun C., Xu J., Li S., Cui M., Fu X., Liu Y., Liu Y., Wan H., Wei K. (2022). Synthesis-on-substrate
of quantum dot solids. Nature.

[8] Kim J. S., Heo J.-M., Park G.-S., Woo S.-J., Cho C., Yun H. J., Kim D.-H., Park J., Lee S.-C., Park S.-H. (2022). Ultra-bright, efficient and stable perovskite light-emitting
diodes. Nature.

[9] Utzat H., Sun W., Kaplan A. E. K., Krieg F., Ginterseder M., Spokoyny B., Klein N. D., Shulenberger K. E., Perkinson C. F., Kovalenko M. V. (2019). Coherent single-photon
emission from colloidal lead halide perovskite quantum dots. Science.

[10] Rainò G., Utzat H., Bawendi M. G., Kovalenko M. V. (2020). Superradiant
emission from self-assembled light emitters: From molecules to quantum
dots. MRS Bull..

[11] Zhu C. L., Boehme S. C., Feld L. G., Moskalenko A., Dirin D. N., Mahrt R. F., Stöferle T., Bodnarchuk M. I., Efros A. L., Sercel P. C. (2024). Single-photon
superradiance in individual caesium lead halide quantum dots. Nature.

[12] Rainò G., Becker M. A., Bodnarchuk M. I., Mahrt R. F., Kovalenko M. V., Stöferle T. (2018). Superfluorescence
from lead halide perovskite quantum
dot superlattices. Nature.

[13] Kagan C. R., Bassett L. C., Murray C. B., Thompson S. M. (2021). Colloidal Quantum
Dots as Platforms for Quantum Information Science. Chem. Rev..

[14] García
de Arquer F. P., Talapin D. V., Klimov V. I., Arakawa Y., Bayer M., Sargent E. H. (2021). Semiconductor quantum dots: Technological
progress and future challenges. Science.

[15] Akkerman Q. A., Nguyen T. P. T., Boehme S. C., Montanarella F., Dirin D. N., Wechsler P., Beiglböck F., Rainò G., Erni R., Katan C. (2022). Controlling
the nucleation and growth kinetics of lead halide perovskite quantum
dots. Science.

[16] Protesescu L., Yakunin S., Nazarenko O., Dirin D. N., Kovalenko M. V. (2018). Low-Cost
Synthesis of Highly Luminescent Colloidal Lead Halide Perovskite Nanocrystals
by Wet Ball Milling. ACS Appl. Nano Mater..

[17] Lignos I., Stavrakis S., Nedelcu G., Protesescu L., Demello A. J., Kovalenko M. V. (2016). Synthesis of Cesium Lead Halide Perovskite
Nanocrystals in a Droplet-Based Microfluidic Platform: Fast Parametric
Space Mapping. Nano Lett..

[18] Display Week 2023 Highlights. Information Display. 2023. 10.1002/msid.0050040 (accessed 03.06.2025).

[19] Bodnarchuk M. I., Boehme S. C., ten Brinck S., Bernasconi C., Shynkarenko Y., Krieg F., Widmer R., Aeschlimann B., Günther D., Kovalenko M. V. (2019). Rationalizing and Controlling
the Surface Structure and Electronic Passivation of Cesium Lead Halide
Nanocrystals. ACS Energy Lett..

[20] Morad V., Stelmakh A., Svyrydenko M., Feld L. G., Boehme S. C., Aebli M., Affolter J., Kaul C. J., Schrenker N. J., Bals S. (2024). Designer phospholipid
capping ligands for soft metal halide nanocrystals. Nature.

[21] Bertolotti F., Protesescu L., Kovalenko M. V., Yakunin S., Cervellino A., Billinge S. J. L., Terban M. W., Pedersen J. S., Masciocchi N., Guagliardi A. (2017). Coherent Nanotwins
and Dynamic Disorder in Cesium Lead
Halide Perovskite Nanocrystals. ACS Nano.

[22] Seiler H., Zahn D., Taylor V. C. A., Bodnarchuk M. I., Windsor Y. W., Kovalenko M. V., Ernstorfer R. (2023). Direct Observation
of Ultrafast Lattice Distortions during Exciton-Polaron Formation
in Lead Halide Perovskite Nanocrystals. ACS
Nano.

[23] Aebli M., Kaul C. J., Yazdani N., Krieg F., Bernasconi C., Guggisberg D., Marczak M., Morad V., Piveteau L., Bodnarchuk M. I. (2024). Disorder and Halide Distributions in Cesium
Lead Halide Nanocrystals as Seen by Colloidal ^133^Cs Nuclear
Magnetic Resonance Spectroscopy. Chem. Mater..

[24] Gallop N. P., Maslennikov D. R., Mondal N., Goetz K. P., Dai Z., Schankler A. M., Sung W., Nihonyanagi S., Tahara T., Bodnarchuk M. I. (2024). Ultrafast vibrational
control of organohalide perovskite optoelectronic devices using vibrationally
promoted electronic resonance. Nat. Mater..

[25] Yazdani N., Bodnarchuk M. I., Bertolotti F., Masciocchi N., Fureraj I., Guzelturk B., Cotts B. L., Zajac M., Rainò G., Jansen M. (2024). Coupling to octahedral
tilts in halide perovskite nanocrystals induces phonon-mediated attractive
interactions between excitons. Nat. Phys..

[26] Nedelcu G., Protesescu L., Yakunin S., Bodnarchuk M. I., Grotevent M. J., Kovalenko M. V. (2015). Fast Anion-Exchange in Highly Luminescent
Nanocrystals of Cesium Lead Halide Perovskites (CsPbX_3_,
X = Cl, Br, I). Nano Lett..

[27] Protesescu L., Yakunin S., Bodnarchuk M. I., Krieg F., Caputo R., Hendon C. H., Yang R. X., Walsh A., Kovalenko M. V. (2015). Nanocrystals
of Cesium Lead Halide Perovskites (CsPbX_3_, X = Cl, Br,
and I): Novel Optoelectronic Materials Showing Bright Emission with
Wide Color Gamut. Nano Lett..

[28] Protesescu L., Yakunin S., Bodnarchuk M. I., Bertolotti F., Masciocchi N., Guagliardi A., Kovalenko M. V. (2016). Monodisperse
Formamidinium Lead Bromide Nanocrystals with Bright and Stable Green
Photoluminescence. J. Am. Chem. Soc..

[29] Protesescu L., Yakunin S., Kumar S., Bär J., Bertolotti F., Masciocchi N., Guagliardi A., Grotevent M., Shorubalko I., Bodnarchuk M. I. (2017). Dismantling the ″Red Wall″ of Colloidal Perovskites:
Highly Luminescent Formamidinium and Formamidinium-Cesium Lead Iodide
Nanocrystals. ACS Nano.

[30] Bodnarchuk M. I., Feld L. G., Zhu C., Boehme S. C., Bertolotti F., Avaro J., Aebli M., Mir S. H., Masciocchi N., Erni R. (2024). Colloidal
Aziridinium Lead Bromide Quantum Dots. ACS Nano.

[31] Akkerman Q. A., D’Innocenzo V., Accornero S., Scarpellini A., Petrozza A., Prato M., Manna L. (2015). Tuning the optical
properties of cesium lead halide perovskite nanocrystals by anion
exchange reactions. J. Am. Chem. Soc..

[32] Rainò G., Yazdani N., Boehme S. C., Kober-Czerny M., Zhu C. L., Krieg F., Rossell M. D., Erni R., Wood V., Infante I. (2022). Ultra-narrow room-temperature emission
from single CsPbBr_3_ perovskite quantum dots. Nat. Commun..

[33] Becker M. A., Vaxenburg R., Nedelcu G., Sercel P. C., Shabaev A., Mehl M. J., Michopoulos J. G., Lambrakos S. G., Bernstein N., Lyons J. L. (2018). Bright
triplet excitons
in caesium lead halide perovskites. Nature.

[34] Dirin D. N., Kovalenko M. V. (2024). The First
Decade of Colloidal Lead Halide Perovskite
Quantum Dots (in our Laboratory). CHIMIA.

[35] Rosales B. A., Schutt K., Berry J. J., Wheeler L. M. (2023). Leveraging Low-Energy
Structural Thermodynamics in Halide Perovskites. ACS Energy Lett..

[36] Brandt R. E., Stevanović V., Ginley D. S., Buonassisi T. (2015). Identifying
defect-tolerant semiconductors with high minority-carrier lifetimes:
beyond hybrid lead halide perovskites. MRS Commun..

[37] Dirin D. N., Protesescu L., Trummer D., Kochetygov I. V., Yakunin S., Krumeich F., Stadie N. P., Kovalenko M. V. (2016). Harnessing
Defect-Tolerance at the Nanoscale: Highly Luminescent Lead Halide
Perovskite Nanocrystals in Mesoporous Silica Matrixes. Nano Lett..

[38] De
Roo J., Ibáñez M., Geiregat P., Nedelcu G., Walravens W., Maes J., Martins J. C., Van Driessche I., Kovalenko M. V., Hens Z. (2016). Highly Dynamic Ligand Binding and
Light Absorption Coefficient of Cesium Lead Bromide Perovskite Nanocrystals. ACS Nano.

[39] Stelmakh A., Aebli M., Baumketner A., Kovalenko M. V. (2021). On the
Mechanism of Alkylammonium Ligands Binding to the Surface of CsPbBr_3_ Nanocrystals. Chem. Mater..

[40] Krieg F., Ochsenbein S. T., Yakunin S., ten Brinck S., Aellen P., Süess A., Clerc B., Guggisberg D., Nazarenko O., Shynkarenko Y. (2018). Colloidal CsPbX_3_ (X = Cl, Br, I) Nanocrystals 2.0: Zwitterionic Capping Ligands
for Improved Durability and Stability. ACS Energy
Lett..

[41] Tan Y., Zou Y., Wu L., Huang Q., Yang D., Chen M., Ban M., Wu C., Wu T., Bai S. (2018). Highly
Luminescent and Stable Perovskite Nanocrystals with Octylphosphonic
Acid as a Ligand for Efficient Light-Emitting Diodes. ACS Appl. Mater. Interfaces.

[42] Almeida G., Infante I., Manna L. (2019). Resurfacing
halide perovskite nanocrystals. Science.

[43] Krieg F., Ong Q. K., Burian M., Rainò G., Naumenko D., Amenitsch H., Süess A., Grotevent M. J., Krumeich F., Bodnarchuk M. I. (2019). Stable Ultraconcentrated and Ultradilute Colloids of CsPbX_3_ (X = Cl, Br) Nanocrystals Using Natural Lecithin as a Capping Ligand. J. Am. Chem. Soc..

[44] Smock S. R., Chen Y., Rossini A. J., Brutchey R. L. (2021). The Surface Chemistry
and Structure of Colloidal Lead Halide Perovskite Nanocrystals. Acc. Chem. Res..

[45] Morad V., Stelmakh A., Svyrydenko M., Feld L. G., Boehme S. C., Aebli M., Affolter J., Kaul C. J., Schrenker N. J., Bals S. (2024). Designer
phospholipid capping ligands for soft metal
halide nanocrystals. Nature.

[46] De
Trizio L., Infante I., Manna L. (2023). Surface Chemistry of
Lead Halide Perovskite Colloidal Nanocrystals. Acc. Chem. Res..

[47] Chen Y., Smock S. R., Flintgruber A. H., Perras F. A., Brutchey R. L., Rossini A. J. (2020). Surface Termination
of CsPbBr_3_ Perovskite
Quantum Dots Determined by Solid-State NMR Spectroscopy. J. Am. Chem. Soc..

[48] Pan A., He B., Fan X., Liu Z., Urban J. J., Alivisatos A. P., He L., Liu Y. (2016). Insight into
the Ligand-Mediated Synthesis of Colloidal
CsPbBr_3_ Perovskite Nanocrystals: The Role of Organic Acid,
Base, and Cesium Precursors. ACS Nano.

[49] Liu Y., Li D., Zhang L., Chen Y., Geng C., Shi S., Zhang Z., Bi W., Xu S. (2020). Amine- and Acid-Free
Synthesis of Stable CsPbBr_3_ Perovskite Nanocrystals. Chem. Mater..

[50] Nenon D. P., Pressler K., Kang J., Koscher B. A., Olshansky J. H., Osowiecki W. T., Koc M. A., Wang L.-W., Alivisatos A. P. (2018). Design
Principles for Trap-Free CsPbX_3_ Nanocrystals: Enumerating
and Eliminating Surface Halide Vacancies with Softer Lewis Bases. J. Am. Chem. Soc..

[51] Zhang B., Goldoni L., Zito J., Dang Z., Almeida G., Zaccaria F., de Wit J., Infante I., De Trizio L., Manna L. (2019). Alkyl Phosphonic Acids
Deliver CsPbBr_3_ Nanocrystals with
High Photoluminescence Quantum Yield and Truncated Octahedron Shape. Chem. Mater..

[52] Yang D., Li X., Zhou W., Zhang S., Meng C., Wu Y., Wang Y., Zeng H. (2019). CsPbBr_3_ Quantum Dots 2.0:
Benzenesulfonic Acid Equivalent Ligand Awakens Complete Purification. Adv. Mater..

[53] Ravi V. K., Santra P. K., Joshi N., Chugh J., Singh S. K., Rensmo H., Ghosh P., Nag A. (2017). Origin of the Substitution
Mechanism for the Binding of Organic Ligands on the Surface of CsPbBr_3_ Perovskite Nanocubes. J. Phys. Chem.
Lett..

[54] Ginterseder M., Sun W., Shcherbakov-Wu W., McIsaac A. R., Berkinsky D. B., Kaplan A. E. K., Wang L., Krajewska C., Šverko T., Perkinson C. F. (2023). Lead Halide Perovskite
Nanocrystals with Low Inhomogeneous Broadening and High Coherent Fraction
through Dicationic Ligand Engineering. Nano
Lett..

[55] Cai Y., Li W., Tian D., Shi S., Chen X., Gao P., Xie R.-J. (2022). Organic Sulfonium-Stabilized High-Efficiency Cesium
or Methylammonium Lead Bromide Perovskite Nanocrystals. Angew. Chem., Int. Ed..

[56] Pegu M., Roshan H., Otero-Martínez C., Goldoni L., Zito J., Livakas N., Rusch P., De Boni F., Stasio F. D., Infante I. (2025). Improving
the Stability
of Colloidal CsPbBr_3_ Nanocrystals with an Alkylphosphonium
Bromide as Surface Ligand Pair. ACS Energy Lett..

[57] Krieg F., Sercel P. C., Burian M., Andrusiv H., Bodnarchuk M. I., Stöferle T., Mahrt R. F., Naumenko D., Amenitsch H., Rainò G. (2021). Monodisperse Long-Chain Sulfobetaine-Capped
CsPbBr_3_ Nanocrystals and Their Superfluorescent Assemblies. ACS Cent. Sci..

[58] Scharf E., Krieg F., Elimelech O., Oded M., Levi A., Dirin D. N., Kovalenko M. V., Banin U. (2022). Ligands Mediate Anion
Exchange between Colloidal Lead-Halide Perovskite Nanocrystals. Nano Lett..

[59] Gallagher S., Kline J., Jahanbakhshi F., Sadighian J. C., Lyons I., Shen G., Hammel B. F., Yazdi S., Dukovic G., Rappe A. M. (2024). Ligand
Equilibrium Influences
Photoluminescence Blinking in CsPbBr_3_: A Change Point Analysis
of Widefield Imaging Data. ACS Nano.

[60] Xu B., Jacobs M. I., Kostko O., Ahmed M. (2017). Guanidinium Group Remains
Protonated in a Strongly Basic Arginine Solution. ChemPhysChem.

[61] Jodlowski A. D., Roldán-Carmona C., Grancini G., Salado M., Ralaiarisoa M., Ahmad S., Koch N., Camacho L., de Miguel G., Nazeeruddin M. K. (2017). Large guanidinium cation mixed with
methylammonium in lead iodide perovskites for 19% efficient solar
cells. Nat. Energy.

[62] Nazarenko O., Kotyrba M. R., Wörle M., Cuervo-Reyes E., Yakunin S., Kovalenko M. V. (2017). Luminescent
and Photoconductive Layered
Lead Halide Perovskite Compounds Comprising Mixtures of Cesium and
Guanidinium Cations. Inorg. Chem..

[63] Wu P., Li D., Wang S., Zhang F. (2023). Magic guanidinium cations in perovskite
solar cells: from bulk to interface. Mater.
Chem. Front..

[64] Aoyagi N., Furusho Y., Endo T. (2014). Convenient Synthesis of Acyclic Guanidines
from Isothiouronium Iodides and Amines without Protection of the Amino
Groups. Synlett.

[65] John R. A., Demiraǧ Y., Shynkarenko Y., Berezovska Y., Ohannessian N., Payvand M., Zeng P., Bodnarchuk M. I., Krumeich F., Kara G. (2022). Reconfigurable halide
perovskite nanocrystal memristors for neuromorphic computing. Nat. Commun..

[66] Tamarat P., Prin E., Berezovska Y., Moskalenko A., Nguyen T. P. T., Xia C., Hou L., Trebbia J.-B., Zacharias M., Pedesseau L. (2023). Universal scaling laws
for charge-carrier interactions with quantum confinement in lead-halide
perovskites. Nat. Commun..

[67] Bertolotti F., Dengo N., Cervellino A., Bodnarchuk M. I., Bernasconi C., Cherniukh I., Berezovska Y., Boehme S. C., Kovalenko M. V., Masciocchi N. (2024). Size- and Temperature-Dependent Lattice Anisotropy
and Structural
Distortion in CsPbBr_3_ Quantum Dots by Reciprocal Space
X-ray Total Scattering Analysis. Small Struct..

[68] Mir W. J., Alamoudi A., Yin J., Yorov K. E., Maity P., Naphade R., Shao B., Wang J., Lintangpradipto M. N., Nematulloev S. (2022). Lecithin Capping Ligands Enable Ultrastable
Perovskite-Phase CsPbI_3_ Quantum Dots for Rec. 2020 Bright-Red
Light-Emitting Diodes. J. Am. Chem. Soc..

[69] Wang C., Chesman A. S. R., Jasieniak J. J. (2017). Stabilizing the cubic perovskite
phase of CsPbI_3_ nanocrystals by using an alkyl phosphinic
acid. Chem. Commun..

[70] Pan J., Shang Y., Yin J., De Bastiani M., Peng W., Dursun I., Sinatra L., El-Zohry A. M., Hedhili M. N., Emwas A.-H. (2018). Bidentate
Ligand-Passivated
CsPbI_3_ Perovskite Nanocrystals for Stable Near-Unity Photoluminescence
Quantum Yield and Efficient Red Light-Emitting Diodes. J. Am. Chem. Soc..

[71] Kobiyama E., Urbonas D., Aymoz B., Bodnarchuk M. I., Rainò G., Olziersky A., Caimi D., Sousa M., Mahrt R. F., Kovalenko M. V. (2025). Perovskite Nanocrystal
Self-Assemblies in 3D Hollow Templates. ACS
Nano.

[72] Morris, G. A. Diffusion-Ordered Spectroscopy. In eMagRes., 2009. 10.1002/9780470034590.emrstm0119.pub2

[73] Cabrita E. J., Berger S. (2002). HR-DOSY as a new tool
for the study of chemical exchange
phenomena. Magn. Reson. Chem..

[74] Sarkar D., Stelmakh A., Karmakar A., Aebli M., Krieg F., Bhattacharya A., Pawsey S., Kovalenko M. V., Michaelis V. K. (2024). Surface
Structure of Lecithin-Capped Cesium Lead Halide
Perovskite Nanocrystals Using Solid-State and Dynamic Nuclear Polarization
NMR Spectroscopy. ACS Nano.

[75] Nichols J. W. (1985). Thermodynamics
and kinetics of phospholipid monomer-vesicle interaction. Biochemistry.

[76] Roseman M. A., Thompson T. E. (1980). Mechanism of the
spontaneous transfer of phospholipids
between bilayers. Biochemistry.

[77] Richens J. L., Tyler A. I. I., Barriga H. M. G., Bramble J. P., Law R. V., Brooks N. J., Seddon J. M., Ces O., O’Shea P. (2017). Spontaneous
charged lipid transfer between lipid vesicles. Sci. Rep..

[78] Amberg W. M., Lindner H., Sahin Y., Staudinger E., Morad V., Sabisch S., Feld L. G., Li Y., Dirin D. N., Kovalenko M. V. (2025). Ligand Influence on
the Performance of Cesium Lead Bromide Perovskite Quantum Dots in
Photocatalytic C­(sp3)-H Bromination Reactions. J. Am. Chem. Soc..

[79] Rosa-Pardo I., Casadevall C., Schmidt L., Claros M., Galian R. E., Lloret-Fillol J., Pérez-Prieto J. (2020). The synergy between the CsPbBr_3_ nanoparticle
surface and the organic ligand becomes manifest
in a demanding carbon-carbon coupling reaction. Chem. Commun..

