# Supplementary material for: Tightly yet Dynamically Bound Aliphatic Guanidinium Ligands for Lead Halide Perovskite Nanocrystals
Source: J Am Chem Soc. 2025 Sep 17;147(39):35446–55. doi: 10.1021/jacs.5c09354 (PMC12498395; doi:10.1021/jacs.5c09354)
Supplement: Supplementary file 1 [file ja5c09354_si_001.pdf]

Supplementary Information for

Tightly yet Dynamically Bound Aliphatic Guanidinium Ligands for Lead Halide Perovskite Nanocrystals

Yuliia Berezovska,[a,b] Sebastian Sabisch,[a,b] Caterina Bernasconi,[a,b] Yesim Sahin,[a,b] Federica Bertolotti,[c] Antonietta Guagliardi,[d] Maryna I. Bodnarchuk,[a,b]* Dmitry N. Dirin,[a,b]* Maksym V. Kovalenko[a,b]*

[a] Institute of Inorganic Chemistry, Department of Chemistry and Applied Biosciences, ETH Zürich, CH-8093 Zürich, Switzerland

[b] Empa–Swiss Federal Laboratories for Materials Science and Technology, CH-8600 Dubendorf, Switzerland

[c] Dipartimento di Scienza e Alta Tecnologia & To.Sca.Lab, Universitadell'Insubria, 22100 Como, Italy

[d] Istituto di Cristallografia & To.Sca.Lab, Consiglio Nazionale delle Ricerche, 22100 Como, Italy

Contents

1. Chemicals ... 4
2. Synthesis of $CsPbBr_3$ NCs capped with the commonly employed ligands ... 5
2.1. Hot-injection synthesis of OAm/OAcH-capped $CsPbBr_3$ NCs ... 5
2.2. Hot-injection synthesis of DDAB-capped $CsPbBr_3$ NCs ... 5
2.3. Hot-injection synthesis of ASC18-capped $CsPbBr_3$ NCs ... 6
2.4. Room temperature synthesis of PC- and PEA-capped $CsPbBr_3$ NCs ... 7
3. Synthesis of guanidinium-based ligands ... 9
3.1. Guanidinylation of amines (Path 1) ... 9
3.2. Functionalization of L-arginine (Path 2) ... 10
4. Synthesis of LHP NCs capped with guanidinium-based ligands ... 11
4.1. Hot-injection synthesis of 8-11 nm large $CsPbBr_3$ NCs capped with guanidinium-based ligands ... 11
4.2. Hot-injection synthesis of 16 nm large LAD-capped $CsPbBr_3$ NCs ... 11
4.3. Hot-injection synthesis of 20 nm large OGB-capped $FAPbBr_3$ NCs ... 11
4.4. Hot-injection synthesis of 5 nm large OGB-capped $CsPbBr_3$ NCs ... 12
4.5. Hot-injection synthesis of 20 nm large OGI-capped $CsPbI_3$ NCs ... 12
4.6 Room temperature synthesis of guanidinium-capped $CsPbBr_3$ NCs ... 12
5. Characterization methods ... 14
5.1. Nuclear Magnetic Resonance Spectroscopy (NMR) ... 14
5.2. Mass Spectrometry (MS) ... 15
5.3. Transmission Electron Microscopy (TEM) ... 16
5.4. UV-Vis optical absorption spectroscopy ... 16
5.5. Photoluminescence (PL) and absolute PL Quantum Yield (QY) measurements ... 16
5.6. Total X-ray scattering ... 16
6. Computational study of the surface fitness of ligands ... 17
7. Photocatalytic performance of $CsPbBr_3$ NCs capped with various ligands ... 18
8. Supporting Figures ... 19
9. Supporting Tables ... 28
10. Supporting information references ... 30

1. Chemicals

All chemicals were used as supplied without further purification.

Oleic acid (OAcH, 90%), 3-(N, N-dimethyloctadecylammonio) propanesulfonate (ASC18, ≥99.0%), phosphorus (V) oxychloride (99%), triethylamine (99%), 2-aminoethan-1-ol (>=99.0%), acetic acid (>99.8%), 2-octyl-1-dodecanol (97%), thiourea, iodomethane, thionyl bromide (97%), 1-octadecylamine, 1-decanol (99%), phenethylamine (99%), diisooctylphosphinic acid (DOPA, 90%), hexanoic acid (99.5%), cesium carbonate (Cs_2CO_3, 99.9%), lead (**II**) acetate trihydrate ($(CH_3COO)_2$ Pb ×$3H_2O$, ≥99.99%), lead (**II**) oxide (PbO, 99.999% trace metal basis), formamidine acetate (99%), 1-adamantanecarboxylic acid (99%), mesitylene, cyclohexane, butyl acetate, acetone (≥99.5%), tetrahydrofuran (THF, ACS reagent, >=99.5%), 1-octadecene (ODE, 90%), N,N-diisopropylethylamine (DIPEA, 99.5%) were purchased from **Sigma – Aldrich**; trimethylamine (2 M in THF) was purchased from **Fluorochem**; lauroyl chloride (98%), lead (**II**) bromide ($PbBr_2$, 98+%), o-xylene (99% pure), and cyclohexane-d_{12} (99.5 atom% D) were purchased from **Acros**; 1,3,5-triethylbenzene (>90.0%), 2-chloro-2-oxo-1,3,2-dioxaphospholane (COP, >95.0% (GC)(T)), S-ethylisothiourea hydrobromide and L-(+)-arginine were purchased from **TCI**; dodecyl amine (for synthesis, 98%), chloroform (≥99.8%), and acetonitrile from **Merck**; methyl acetate from **ABCR**; cis-9-octadecen-1-ol (for synthesis), sodium hydroxide and diethyl ether from **VWR**; trioctylphosphine (TOP, 97%), trioctylphosphine oxide (TOPO, 90%) and oleylamine (OAm, 95%) from **Strem Chemicals**; octylamine, benzene-d_6 (C_6D_6, 99.5 atom% D) and dimethylsulfoxide-d_6 (DMSO-d_6, 99.8 atom% D) from **Apollo Scientific**; Isopropanol, 1,4-dioxane (99.5% extra dry over molecular sieve), and chloroform-d ($CDCl_3$, 99.8 atom% D) were purchased from **Fisher Scientific;** lecithin (from soy, >97%) and n-octane (≥99%) were purchased from **Roth**; ethanol absolute and benzyl bromide were purchased from **Fluka.**

2. Synthesis of $CsPbBr_3$ NCs capped with the commonly employed ligands

The initial survey of ligand binding dynamics was performed for the most commonly employed ligands featuring primary ammonium (OAm/OAcH), quaternary ammonium (DDAB), sulfobetaine (ASC18), phosphocholine (PC), and phosphoethanolamine (PEA) head groups. Conventional synthetic and washing protocols were used in all cases.

2.1. Hot-injection synthesis of OAm/OAcH-capped $CsPbBr_3$ NCs

$CsPbBr_3$ NCs capped with oleylamine and oleic acid (Oam/OAcH) were synthesized as described elsewhere with slight modifications.[1]

Synthesis

Cesium oleate (Cs-oleate) solution was prepared by mixing Cs_2CO_3 (0.814 g) with oleic acid (2.5 mL) in a three-neck flask along with 40 mL of ODE at room temperature under vacuum, until the first gas evolution stops and then further heated to 120°C for 1 hour. This yields a 0.4 M solution of Cs-oleate in ODE. The solution was stored under nitrogen and heated before use.

$PbBr_2$ (69.5 mg) and ODE (5 mL) were loaded into a 25 mL three-necked flask and dried under vacuum for 30 min at 110°C. The resulting mixture was first heated to 150°C. When the reaction mixture reached 150°C, dried OAcH (0.5 mL) was added, and the reaction mixture further heated to 180°C. After the $PbBr_2$ dissolved, dried OAm (0.5 mL) was added, and once the mixture reached 180°C again the Cs-oleate solution (0.4 mL) was quickly injected. After 10 seconds, the reaction mixture was rapidly cooled to ~30°C with an ice bath.

Purification

The crude solution was transferred to a centrifuge tube and centrifuged for 5 min at 12.1 krpm (20133×g). Precipitate was redispersed in 0.8 mL of benzene-d_6. The obtained colloidal solution was centrifuged for 3 min at 12.1 krpm (20133×g). 0.9 mL of methyl acetate was added to 0.75 mL of the colloidal solution, followed by centrifugation for 2 min at 13.4 krpm (12100×g). Precipitate was redispersed in 0.75 mL of benzene-d_6. The obtained colloidal solution was centrifuged for 2 min at 13.4 krpm (12100×g) and 0.55 mL of the supernatant was used for NMR studies (1st washing).

0.25 mL of the previously obtained solution was mixed with 0.2 mL of methyl acetate, followed by centrifugation for 2 min at 13.4 krpm (12100×g). Precipitate was redispersed in 0.2 mL of benzene-d_6. The obtained colloidal solution was centrifuged for 90 seconds at 12.1 krpm (20133×g) and 0.5 mL of the supernatant was used for NMR studies (2nd washing).

2.2. Hot-injection synthesis of DDAB-capped $CsPbBr_3$ NCs

$CsPbBr_3$ NCs capped with didodecyldimethylammonium bromide (DDAB) were synthesized as described elsewhere with slight modifications.[2]

Synthesis

Cesium diisooctylphosphinate (Cs-DOPA, 0.144 M) solution was prepared by mixing Cs_2CO_3 (200 mg, 0.614 mmol) with toluene (7.5 mL) and diisooctylphosphinic acid (DOPA, 1 mL, 3.154 mmol) in a vial. The mixture was stirred and heated to 120 °C on a hot plate until it became clear and then cooled down to room temperature.

$PbBr_2$ (55 mg, 0.15 mmol) and DDAB (61 mg, 0.132 mmol) were added to 4 mL of 1,3,5-triethylbenzene in a 25 mL three-necked-flask. With vigorous magnetic stirring, the mixture was heated to 180°C until it became clear, and 0.4 mL of Cs-DOPA solution was swiftly injected using a 1-mL syringe with a 1.6 × 40 mm needle. After 10 seconds, the reaction mixture was cooled down to ~30°C with an ice bath.

Purification

The crude solution was split into fractions of 2 mL, transferred to a centrifuge tube and centrifuged for 2 min at 12.1 krpm (20133×g). Precipitate was dissolved in 2 mL of cyclohexane, and 2 mL of toluene and 4 mL of ethyl acetate were added, followed by centrifugation for 2 min at 12.1 krpm (20133×g). Precipitate was redispersed in 0.4 mL of cyclohexane-d_{12}. The obtained colloidal solution was centrifuged for 1 min at 12.1 krpm (20133×g), and 0.55 mL of the combined supernatant was used for NMR studies (1st washing).

The remaining precipitate was dissolved in 0.5 mL of cyclohexane and combined with the remaining previously obtained supernatant. The resulting solution was mixed with 1.5 mL of toluene and 4 mL of ethyl acetate, followed by centrifugation for 1 min at 12.1 krpm (20133×g). Precipitate was redispersed in 0.4 mL of cyclohexane. The resulting solution was mixed with 0.4 mL of toluene and 1.5 mL of ethyl acetate, followed by centrifugation for 1 min at 12.1 krpm (20133×g). Precipitate was redispersed in 0.4 mL of cyclohexane-d_{12}. The obtained colloidal solution was centrifuged for 1 min at 12.1 krpm (20133×g) and the supernatant was used for NMR studies (3rd washing).

2.3. Hot-injection synthesis of ASC18-capped $CsPbBr_3$ NCs

$CsPbBr_3$ NCs capped with 3-(N, N-dimethyloctadecylammonio) propanesulfonate (an ammonio sulfonate with a C_{18} alkyl tail, herein denoted as ASC18) were synthesized as described elsewhere with slight modifications.[3]

Synthesis

Cesium oleate (Cs-oleate) solution was prepared by mixing Cs_2CO3 (1.628 g, 5 mmol, containing 10 mmol Cs, i.e. 1 eq) and oleic acid (5 mL, 16 mmol, 1.6 eq) and evacuated in a three-neck flask along with 20 mL of ODE at room temperature until the first gas evolution stops and then was further evacuated at 120°C for 1 hour. This yields a 0.4 M solution of Cs-oleate in ODE. The solution turns solid when cooled to room temperature. It was stored under nitrogen and heated before use.

Lead (II)-oleate (Pb-oleate) solution was prepared by mixing $(CH_3COO)_2$ Pb ×$3H_2O$ (4.6066 g, 12 mmol, 1 eq) and oleic acid (7.6 mL, 24 mmol, 2 eq) in a three-neck flask along with 16.4 mL of ODE at room temperature under vacuum until the first gas evolution stops. Afterwards, the solution was further dried at 120°C for 1 hour. This yields a 0.5 M solution of Pb-oleate in ODE. The solution turns solid when cooled to room temperature. It was stored under nitrogen and heated before use.

$TOPBr_2$ solution was prepared by mixing TOP (6 mL, 13 mmol, 1 eq) with Br_2 (0.6 mL, 11.5 mmol, 0.88 eq). The reaction between the two components is exothermic and requires vigorous stirring due to the product being highly viscous (white, almost solid). It was dissolved in toluene (18.7 mL) after a complete reaction to create a 0.46 M light yellow stock solution. The reaction was carried out in a Schlenk flask under nitrogen.

ASC18 (63 mg) and 5 mL of ODE were loaded in a 25 mL three-necked flask along with preheated Pb-oleate (1.5 mL) and Cs-oleate (1.2 mL) precursors. The reaction vessel was purged 3 times and heated up to 130°C under a nitrogen atmosphere. After reaching the reaction temperature, $TOPBr_2$ solution (1.5 mL) was injected. After the injection, the reaction mixture was immediately cooled down to room temperature with an ice bath.

Purification

The crude solution was transferred to a centrifuge tube and centrifuged for 10 min at 12.1 krpm (20133×g). Remaining precipitate was discarded. 3 mL of toluene and 22 mL of ethyl acetate were added to the resulting supernatant, followed by centrifugation for 10 min at 12.1 krpm (20133×g). Precipitate was redispersed in 1.2 mL of benzene-d_6. The obtained colloidal solution was centrifuged for 3 min at 12.1 krpm (20133×g) and 0.55 mL of the supernatant was used for NMR studies (1st washing).

0.85 mL of the previously obtained solution was mixed with 2 mL of ethyl acetate, followed by centrifugation for 1 min at 12.1 krpm (20133×g). Precipitate was redispersed in 1 mL of benzene-d_6. The obtained colloidal solution was mixed with 2 mL of ethyl acetate, followed by centrifugation for 1 min at 12.1 krpm (20133×g). Precipitate was redispersed in 0.5 mL of benzene-d_6. The obtained colloidal solution was centrifuged for 3 min at 12.1 krpm (20133×g) and the supernatant was used for NMR studies (3rd washing).

2.4. Room temperature synthesis of PC- and PEA-capped $CsPbBr_3$ NCs

$CsPbBr_3$ NCs capped with ligands featuring phosphocholine (PC) and phosphoethanolamine (PEA) head groups were synthesized according to the modified procedure described earlier.[4] All PEA- and PC-based ligands were synthesized according to the procedure described in our earlier report.[5]

Synthesis of ligands

PEA ligand synthesis. Solution of alkyl alcohol (0.025 mol; 1 equiv) dissolved in dry tetrahydrofuran (THF) (25 mL) along with triethylamine (0.275 mol; 1.1 equiv; 3.83 mL) was added dropwise with vigorous stirring to a solution of phosphorous oxychloride (0.03 mol; 1.2 equiv; 2.78 mL) in THF (2.5 mL) previously cooled down with an ice water bath. The reaction mixture was subsequently kept at 20 °C for 15 min to complete the reaction. Next, ethanolamine (0.03 mol; 1.2 equiv; 1.81 mL) and triethylamine (0.06 mol; 2.4 equiv) in THF (37.5 mL) were added dropwise under vigorous stirring to the reaction mixture kept in a room-temperature water bath. Subsequently, the mixture was heated to 40 °C for 15 min to complete the ring closure. Finally, the reaction mixture was filtered to remove precipitated triethylamine hydrochloride, and the filtrate solution was dried. An oily residue of alkyl-2-oxo-1,2,3-oxazaphospholane was dissolved in a mixture of acetic acid (5.7 mL) and distilled water (2.6 mL) at 70 °C. After 30 min, ring scission at the P–N bond is complete, and the product is separated by shaking with acetone (125–150 mL). After cooling to 10 °C, alkylphosphoethanolamine is collected and dried overnight under a vacuum at 40–50 °C.

2-Ammonioethyl 2-octyl-1-dodecyl phosphate (branched PEA, brPEA) was synthesized starting from 2-octyl-1-dodecanol. NMR spectra are in agreement with the literature.

2-Ammonioethyl oleyl phosphate (OPEA) was synthesized starting from oleyl alcohol. NMR spectra are in agreement with the literature.

PC ligand synthesis. A solution of 2-chloro-2-oxo-1,3,2-dioxaphospholane (COP)(0.7 mol; 1equiv; 10 g) in dry THF (30 mL) was added dropwise to a mixture of alkyl alcohol substrate (0.7 mol; 1 equiv) and triethylamine (0.7 mol; 1 equiv) in dry THF (140 mL) at 0 °C under vigorous stirring. After the addition, stirring was continued for 1 h at room temperature. After filtering, the filtrate solution was concentrated by at least a factor of two by evaporation. Dry acetonitrile (150 mL) was added, and the reaction mixture was placed into a glass pressure bottle. At −20 °C, 2 M trimethylamine in THF (0.14 mol; 2 equiv; 70 mL) was added and the reaction was carried out at 70 °C for 12 h. After 12 h, the reaction mixture was cooled to −20 °C to precipitate the product. The product was then filtered off and dried under a vacuum overnight.

(2-(Trimethylammonio)ethyl) oleyl phosphate (OPC) was synthesized starting from oleyl alcohol. NMR spectra are in agreement with the literature.

Synthesis of NCs

$PbBr_2$ precursor solution (0.04 M) was prepared from $PbBr_2$ (1 mmol, 367 mg) and TOPO (90%) (5 mmol, 1.93 g) dissolved in n-octane (5 mL) at 120 °C on a hotplate under ambient conditions. Once all the $PbBr_2$ was dissolved, the vial was cooled to room temperature, diluted by 20 mL of hexane. The resulting solution was filtered through a 0.2 µl PTFE filter and stored under ambient conditions.

Cs-DOPA precursor solution (0.02 M) was prepared by loading 100 mg of Cs_2CO_3 together with 1 mL of DOPA and 2 mL of octane at 120 °C in a vial. Once all the Cs_2CO_3 was dissolved, the stock solution was cooled to room temperature, and 27 mL of hexane was added. The resulting solution was filtered through a 0.2 µl PTFE filter and stored under ambient conditions.

Lecithin precursor solution (0.13 M) was prepared by dissolving 0.5 g of lecithin in 10 mL of hexane. The solution was sonicated until the complete dissolution of lecithin, filtered through a 0.2 µl PTFE filter, and stored under ambient conditions.

PEA precursor solution (0.1 mg/µL) was prepared by dissolving 100 mg of PEA ligand in 1 mL of solvent. The solution was left stirring until the complete dissolution of the organic compound. brPEA was dissolved in mesitylene, oleate PEA (OPEA) - in $CHCl_3$.

PC precursor solution (0.1 mg/µL) was prepared by dissolving 100 mg of oleate PC (OPC) ligand in 1 mL of $CHCl_3$. The solution was left stirring until the complete dissolution of OPC.

For the synthesis of $CsPbBr_3$ NCs, 2 mL of Cs-DOPA precursor is swiftly injected into the mixture of 6 mL of hexane and 4 mL of $PbBr_2$ precursor under vigorous stirring at room temperature and left for 10 min to nucleate and grow NCs, followed by the addition of the ligand solution (the amount is specified in the table below) and stirring for 1 minute to ensure ligand exchange.

ligand	lecithin	brPEA	OPEA	OPC
added amount of ligand solution	1 mL	0.3 mL	0.25 mL	0.25 mL
deuterated solvent used for NMR studies	benzene-d_6	cyclohexane-d_{12}	benzene-d_6	benzene-d_6

Purification

Once washed. 20 mL of acetone was added to the crude solution, followed by centrifugation for 2 min at 12.1 krpm (20133×g). Precipitate was redispersed in 0.6 mL of deuterated solvent. The obtained colloidal solution was centrifuged for 3 min at 12.1 krpm (20133×g) and 0.55 mL of the supernatant was used for NMR studies.

Thrice washed. 20 mL of acetone was added to the crude solution, followed by centrifugation for 2 min at 12.1 krpm (20133×g). Precipitate was redispersed in 0.6 mL of non-deuterated solvent. The obtained colloidal solution was mixed with 3 mL of acetone, followed by centrifugation for 2 min at 12.1 krpm (20133×g). Precipitate was redispersed in 0.6 mL of non-deuterated solvent. The obtained colloidal solution was mixed with 2 mL of acetone, followed by centrifugation for 2 min at 12.1 krpm (20133×g). Precipitate was redispersed in 0.5 mL of deuterated solvent. The obtained colloidal solution was centrifuged for 1 min at 12.1 krpm (20133×g), and the supernatant was used for NMR studies.

3. Synthesis of guanidinium-based ligands

3.1. Guanidinylation of amines (Path 1)

Synthesis was performed adapting earlier literature.[6]

Commercial S-ethylisothiourea hydrobromide was used for the synthesis of guanidinium bromide salts. S-methylisothiourea hydroiodide was used for the synthesis of guanidinium iodide salts and was prepared as follows. A solution of methyl iodide (1.2 eq) in 5mL of ethanol was added dropwise to a suspension of thiourea (1 eq) in absolute ethanol (0.1 g/mL). The resulting mixture was stirred overnight at room temperature, and then ethanol was evaporated under vacuum. The resulting solid residue was washed a couple of times with Et_2O and dried under reduced pressure overnight at 50°C. The product was obtained as white crystals with a 99% yield.

Respective S-alkylisothiourea hydrohalide (1.2 eq) was suspended in THF (0.1 g/mL), and amine (1 eq) was added. The resulting mixture was stirred at room temperature overnight. Then, THF was evaporated under reduced pressure, and the resulting residue was recrystallized with Et_2O. The precipitate obtained was washed a couple of times with Et_2O and dried under reduced pressure overnight at 50°C.

The following salts were prepared according to this method.

N-octyl guanidinium bromide (**1, OctGB**). Yield: 96%. Appearance: white powder. ^{1}H NMR (300 MHz, DMSO-*d6*) δ, ppm (J, Hz): 7.49 (t, *J* = 5.7 Hz, 1H), 6.99 (s, 4H), 3.09 (q, *J* = 6.8 Hz, 2H), 1.45 (p, *J* = 7.5 Hz, 2H), 1.25 (t, *J* = 3.6 Hz, 10H), 0.91 – 0.81 (m, 3H). ^{13}C NMR (75 MHz, DMSO-*d6*) δ, ppm: 156.70, 40.69, 31.16, 28.57, 28.51, 28.39, 25.99, 22.03, 13.91. Mass spectrum (m/z) $C_9H_{22}BrN_3$ (EXACT MASS = 252.195) 172.1808 (100%, $[M+H–HBr]^+$).

Phenethyl guanidinium bromide (**2, PhEtGB**). Yield: 53%. Appearance: white powder. ^{1}H NMR (300 MHz, DMSO-*d6*) δ, ppm (J, Hz): 7.56 (t, *J* = 5.7 Hz, 1H), 7.39 – 7.16 (m, 5H), 7.15 – 6.82 (s, 4H), 3.45 – 3.32 (m, 2H), 2.79 (t, *J* = 7.4 Hz, 2H). ^{13}C NMR (75 MHz, DMSO-*d6*) δ, ppm: 156.73, 138.26, 128.77, 128.61, 128.57, 128.33, 126.38, 42.02, 34.40. Mass spectrum (m/z) $C_9H_{14}BrN_3$ (EXACT MASS = 244.13) 164.1181 (100%, $[M+H–HBr]^+$).

N-(2-octyldodecyl) guanidinium bromide (**3, 2-ODGB**). The residue was dissolved in n-BuOH and washed twice with water and afterward with brine solution. The organic layer was dried over Na_2SO_4 and evaporated under reduced pressure. Yield: 76%. Appearance: colorless oil. ^{1}H NMR (400 MHz, DMSO-*d6*) δ, ppm (J, Hz): 8.01 – 6.50 (m, 3H), 3.01 (t, J = 5.8 Hz, 1H), 1.24 (s, 33H), 0.86 (t, J = 6.7 Hz, 6H).

^{13}C NMR (101 MHz, DMSO-*d6*) δ, ppm: 157.86, 44.56, 37.20, 31.79 (s, 2C), 31.04, 29.83 – 29.17 (m, 11C), 26.21, 26.19, 22.58 (s, 2C), 14.39 (s, 2C). Mass spectrum (m/z) $C_{21}H_{46}BrN_3$ (EXACT MASS = 420.514) 340.3681 (100%, $[M+H–HBr]^+$).

N-dodecyl guanidinium bromide (**4, DGB**). Yield: 98%. Appearance: white powder. ^{1}H NMR (400 MHz, DMSO-d6) δ, ppm (J, Hz): 7.48 (s, 1H), 7.05 (d, J = 114.1 Hz, 4H), 3.09 (t, J = 7.1 Hz, 2H), 1.44 (q, J = 6.9 Hz, 2H), 1.27 (d, J = 4.8 Hz, 4H), 1.25 (s, 13H), 0.86 (t, J = 6.7 Hz, 3H). ^{13}C NMR (101 MHz, DMSO-d6) δ, ppm: 157.19, 41.20, 31.77, 29.51, 29.48, 29.45, 29.44, 29.18, 29.07, 28.91, 26.51, 22.56, 14.43. Mass spectrum (m/z) $C_{13}H_{30}BrN_3$ (EXACT MASS = 308.3) 228.2434 (100%, $[M+H–HBr]^+$).

N-(octadec-9-en-1-yl) guanidinium bromide (**5, OGB**). Yield: 56%. Appearance: white powder. ^{1}H NMR (400 MHz, DMSO-*d6*) δ, ppm (J, Hz): δ 7.49 (s, 1H), 7.04 (d, *J* = 140.9 Hz, 4H), 5.44 – 5.19 (m, 2H), 3.09 (q, *J* = 6.6 Hz, 2H), 1.96 (dq, *J* = 17.0, 6.0 Hz, 2H), 1.44 (q, *J* = 7.0 Hz, 2H), 1.25 (d, *J* = 9.6 Hz, 24H), 0.86 (t, *J* = 6.8 Hz, 3H). ^{13}C NMR (101 MHz, DMSO) δ, ppm: 157.19, 130.31 (dd, 2C), 41.21, 32.42, 31.76 (d, 2C), 29.50 (q, 2C), 29.3, 29.17 (d, 2C), 29.08, 28.93, 27.08, 27.04, 26.52, 22.56, 14.41. Mass spectrum (m/z) $C_{19}H_{40}BrN_3$ (EXACT MASS = 390.445) 310.212 (100%, $[M+H–HBr]^+$).

N-octadecyl guanidinium bromide (**6, SGB**). Yield: 99%. Appearance: white powder. ^{1}H NMR (400 MHz, DMSO-d_6) δ, ppm (J, Hz): δ 7.48 (t, J = 5.6 Hz, 1H), 7.40 – 6.53 (m, 4H), 3.09 (q, J = 6.6 Hz, 2H), 1.57 – 1.39 (m, 2H), 1.24 (m, 28H), 0.89 – 0.82 (t, 3H). ^{13}C NMR (101 MHz, DMSO-d_6) δ, ppm: 157.18, 41.21, 39.28, 31.77, 29.52 – 29.41 (m, 9C), 29.18, 29.10, 28.91, 26.52, 22.57, 14.41. Mass spectrum (m/z) $C_{19}H_{42}BrN_3$ (EXACT MASS = 392.46) 312.3372 (100%, $[M+H–HBr]^+$).

N-dodecyl guanidinium iodide (**DGI**). Yield: 89%. Appearance: white powder. ^{1}H NMR (300 MHz, DMSO-d_6) δ, ppm (J, Hz): 7.35 (s, 1H), 6.93 (s, 4H), 3.08 (q, J = 5.9 Hz, 2H), 1.50 – 1.40 (m, 2H), 1.25 (s, 16H), 0.91 – 0.80 (m, 3H). ^{13}C NMR (75 MHz, DMSO-d_6) δ, ppm: 156.56, 40.72, 31.25, 28.99, 28.97, 28.93 (s, 2C), 28.66, 28.55, 28.40, 25.99, 22.05, 13.92. Mass spectrum (m/z) $C_{13}H_{30}IN_3$ (EXACT MASS = 355.3) 228.2445 (100%, $[M+H–HI]^+$).

N-(octadec-9-en-1-yl) guanidinium iodide (**OGI**). Yield: 63%. Appearance: white powder. ^{1}H NMR (400 MHz, DMSO-d_6) δ, ppm (J, Hz): δ 7.32 (s, 1H), 6.97 (s, 4H), 5.34 (s, 2H), 3.07 (s, 2H), 1.96 (s, 2H), 1.44 (s, 2H), 1.24 (s, 24H), 0.85 (s, 3H). ^{13}C NMR (101 MHz, DMSO) δ, ppm: 156.57, 130.04, 129.60, 40.73, 31.90, 31.24, 28.98 (s, 5C), 28.79, 28.65, 28.56, 28.41, 26.55, 26.00, 22.05, 13.91. Mass spectrum (m/z) $C_{19}H_{40}IN_3$ (EXACT MASS = 437.4455) 310.323 (100%, $[M+H–HI]^+$).

3.2. Functionalization of L-arginine (Path 2)

Step 1

Isopropanol (45 mL) and water (15 mL) were added to L-arginine (0.041 mol, 1 eq), , and the mixture was adjusted to a pH of 11 with 27 % aqueous sodium hydroxide solution. While maintaining the temperature at ~ 25 – 27 °C and the pH at 10.5 - 11.5, lauroyl chloride (0.045 mol, 1.1 eq) and 27 % aqueous sodium hydroxide solution (40 mL, 0.045 mol, 1.1 eq) were slowly added dropwise, and the mixture was left stirring at room temperature for 1 hour. Thereafter, the pH was adjusted to 12 with 27 % aqueous sodium hydroxide solution, and the mixture was warmed up to 52 °C. The pH was adjusted to 6 with concentrated hydrochloric acid and then left stirring under heating for half an hour. Afterward, the mixture was cooled down with an ice bath for 1 hr. The obtained suspension was filtered, and the solid collected by filtration was washed with ethyl Acetate to give N2-lauroyl-L-arginine.[7]

Step 2

Synthesis was performed following the literature procedure[8] with some modifications. N2-lauryl-L-arginine (7.12 g, 0.0204 mmol) was suspended in decanol (80 mL), and thionyl bromide (0.051 mol, 2.5 eq) was added dropwise at 0 °C. The mixture was left to stir at room temperature overnight. Afterward, the mixture was distilled under vacuum, and the residue was recrystallized with ethyl acetate to give N2-lauroyl-L-arginine decyl ester hydrobromide (**7, LAD**) as a white solid with a yield of 74%.

N2- lauroyl-L-arginine decyl ester hydrobromide (**7**, **LAD**). ^{1}H NMR (300 MHz, DMSO-d_6) δ, ppm (J, Hz): δ 8.18 (d, J = 7.6 Hz, 1H), 7.48 (t, J = 5.7 Hz, 1H), 6.99 (s, 4H), 4.20 (td, J = 8.3, 5.4 Hz, 1H), 4.02 (tt, J = 6.5, 2.8 Hz, 2H), 3.09 (q, J = 6.6 Hz, 2H), 2.11 (t, J = 7.3 Hz, 2H), 1.85 – 1.45 (m, 8H), 1.38 – 1.10 (m, 30H), 0.91 – 0.81 (m, 6H). ^{13}C NMR (75 MHz, DMSO-d_6) δ, ppm: 172.42, 172.04, 156.61, 64.29, 51.58, 34.95, 31.27(s, 2C), 29.11 – 28.50 (m, 11C), 28.10, 27.84, 25.26 (s, 2C), 25.08, 22.07(s, 2C), 13.91(s, 2C). Mass spectrum (m/z) $C_{28}H_{57}BrN_4O_3$ (EXACT MASS = 577.68) 497.4418 (100%, $[M+H–HBr]^+$).

4. Synthesis of LHP NCs capped with guanidinium-based ligands

4.1. Hot-injection synthesis of 8-11 nm large $CsPbBr_3$ NCs capped with guanidinium-based ligands

Synthesis

$(CH_3COO)_2$ Pb ×$3H_2O$ (49.3 mg, 0.13mmol), cesium carbonate (10.6 mg, 0.0325 mmol), hexanoic acid (0.231 mL, 1.85 mmol), and mesitylene (5 mL) were loaded into a 25mL three-necked flask. Under vigorous stirring, the mixture was heated to 90°C. After reaching the reaction temperature, the guanidinium-based ligand (0.65 mmol) solution in 1 mL of mesitylene was injected using a 3-mL syringe with a 1.6x 40mm needle. After 10 seconds, the reaction mixture was cooled down to room temperature with an ice bath.

Purification

0.3 mL of butyl acetate and 0.15 mL of methyl acetate were added to 0.5 mL of the crude solution, followed by centrifugation for 4 min at 11 krpm (8100×g). The precipitate was discarded. 0.15 mL of methyl acetate was added to the supernatant, followed by centrifugation for 4 min at 11 krpm (8100×g). The precipitate was redispersed in 0.15 mL of cyclohexane. The obtained colloidal solution was centrifuged for 90 sec at 4 krpm (1100×g), and the bright green supernatant was used for further analysis.

LAD-capped $CsPbBr_3$ NCs were purified by a slightly altered procedure. 0.5 mL of the crude solution was centrifuged for 90 sec at 4 krpm (1100×g). Precipitate was discarded. 0.3 mL of butyl acetate and 0.25 mL of methyl acetate were added to the supernatant, followed by centrifugation for 4 min at 11 krpm (8100×g). Precipitate was redispersed in 0.15 mL of cyclohexane. The obtained colloidal solution was centrifuged for 90 sec at 4 krpm (1100×g), and the bright green supernatant was used for further analysis.

4.2. Hot-injection synthesis of 16 nm large LAD-capped $CsPbBr_3$ NCs

Synthesis

NCs were synthesized following the procedure described above (4.1) with slight modifications. $(CH_3COO)_2$ Pb ×$3H_2O$ (49.3 mg, 0.13mmol), cesium carbonate (10.6 mg, 0.0325 mmol), hexanoic acid (0.231 mL, 1.85 mmol) and mesitylene (5 mL) were loaded in a 25mL three-necked flask. Under vigorous stirring, the mixture was heated to 90°C. After reaching the reaction temperature, the Guanidinium-based ligand (0.65 mmol) solution in 1 mL of mesitylene was injected using a 3-mL syringe with a 1.6x 40mm needle. After 105 min, the reaction mixture was cooled down to RT with an ice bath.

Purification

The crude solution was centrifuged for 2 min at 3 krpm (1200×g). The precipitate was redispersed in 4 mL of hexane, followed by centrifugation for 2 min at 3 krpm (1200×g). The green supernatant was set aside, while precipitate was redispersed in 1 mL of hexane and centrifuged for 2 min at 3 krpm (1200×g). The obtained supernatant was combined with the previous one and was further analysed.

4.3. Hot-injection synthesis of 20 nm large OGB-capped $FAPbBr_3$ NCs

Synthesis

OGB precursor (0.2 M in toluene) was prepared by dissolving OGB (156 mg) in 2 mL of toluene, followed by stirring at about 70 °C on a hot plate until complete dissolution.

PbO (9 mg, 0.04 mmol), formamidine acetate (5 mg, 0.04 mmol), and 1-Adamantanecarboxylic acid (165 mg, 0.9 mmol) were loaded into a 25 mL flask. The flask was flushed 3 times with nitrogen. O-xylene (6 mL) was added, and the mixture was heated to about 85 °C until it became transparent; then it was left to cool to 40 °C. After the reaction mixture reached 40 °C, 0.6 mL of the OGB precursor was injected swiftly from a 1 mL syringe. After 5 seconds, the mixture was cooled down to room temperature with an ice bath.

Purification

The crude solution was centrifuged for 3 minutes at 12.1 krpm (20133×g). The precipitate was discarded. 0.7 mL of methyl acetate and 20 µL of 0.02 M solution of OGB in toluene were added to 2 mL of the supernatant, followed by centrifugation for 5 min at 12.1 krpm (20133×g). Precipitate was redispersed in 0.5 mL of cyclohexane. The obtained colloidal solution was centrifuged for 90 sec at 4 krpm (1100×g), and the bright green supernatant was used for further analysis.

4.4. Hot-injection synthesis of 5 nm large OGB-capped $CsPbBr_3$ NCs

Synthesis

PbO (29 mg, 0.13 mmol), cesium carbonate (10.6 mg, 0.0325 mmol), oleic acid (0.231 mL, 1.85 mmol), and mesitylene (5 mL) were loaded in a 25mL three-necked flask. The mixture was heated to 90°C under vigorous stirring. Upon reaching the reaction temperature, OGB (0.65 mmol) solution in 1 mL of mesitylene was injected using a 3-mL syringe with a 1.6x 40mm needle. After 10 s, the reaction mixture was cooled down to room temperature with an ice bath.

Purification

0.2 mL of toluene and 0.2 mL of acetonitrile were added to 0.5 mL of the crude solution, followed by centrifugation for 4 min at 11 krpm (8100×g). Precipitate was redispersed in 0.1 mL of toluene. The obtained colloidal solution was centrifuged for 90 sec at 4 krpm (1100×g), and the supernatant was used for further analysis.

4.5. Hot-injection synthesis of 20 nm large OGI-capped $CsPbI_3$ NCs

Synthesis

$(CH_3COO)_2$ Pb ×3H_2O (49.3 mg, 0.13 mmol), cesium carbonate (10.6 mg, 0.0325 mmol), hexanoic acid (0.231 mL, 1.85 mmol), and mesitylene (5 mL) were loaded in a 25 mL three-necked flask. With vigorous magnetic stirring, the mixture was heated to 90°C. After reaching the reaction temperature, OGI (283 mg, 0.65 mmol) solution in 1 mL of mesitylene was injected using a 3-mL syringe with a 1.6x40 mm needle. After 10 seconds, the reaction mixture was cooled down to room temperature with an ice bath.

Purification

0.5 mL of crude solution was centrifuged for 3 min at 11 krpm (8100×g). The precipitate was dissolved in 0.5 mL of cyclohexane. 0.5 mL of toluene and 0.25 mL of ethyl acetate were added to the colloidal solution, followed by centrifugation for 3 min at 11 krpm (8100×g). The precipitate was redispersed in 0.5 mL of cyclohexane. The obtained colloidal solution was centrifuged for 90 sec at 4 krpm (1100×g), and the dark brown supernatant was used for further analysis.

4.6 Room temperature synthesis of guanidinium-capped $CsPbBr_3$ NCs

NCs were synthesized using the modified procedure described above in Section 2.4.

Synthesis

The OGB precursor (0.13 M) was prepared by dissolving 0.65 mmol (252.5 mg) of OGB in 5 mL of toluene. The solution was heated to dissolve the OGB.

Cs-DOPA precursor was swiftly injected into a mixture of hexane and $PbBr_2$ precursor under vigorous stirring at room temperature and left for a defined amount of time for NC nucleation and growth, followed by the addition of the ligand and stirring for 1 minute to ensure ligand exchange.

NCs size	n-hexane	$PbBr_2$ precursor	Cs-DOPA precursor	Reaction time	OGB
11 nm	3 mL	2 mL	1 mL	10 min	1 mL
9 nm	12 mL	2 mL	1 mL	15 min	1 mL
7 nm	25 mL	2 mL	1 mL	30 min	250 µL (1.3 M)
5 nm	50 mL	2 mL	1 mL	60 min	250 µL (1.3 M)

Purification

The crude solution was concentrated under reduced pressure to ~ 1.5 mL. 4 mL of MeOAC was added to the resulting solution, followed by centrifugation for 2 min at 12.1 krpm (20133×g). The precipitate was redispersed in 1 mL of cyclohexane. The obtained colloidal solution was centrifuged for 1 min at 12.1 krpm (20133×g), and the bright green supernatant was used for further analysis.

5. Characterization methods

5.1. Nuclear Magnetic Resonance Spectroscopy (NMR)

^{1}H spectra for the characterization of the ligands were acquired on an AVANCE III (Bruker) spectrometer at 7 T (300 MHz) using a 5 mm PABBO probe and were referenced to the ^{2}H signal of the respective deuterated solvent. Spectra were recorded with a 30° excitation pulse and a recycle delay of 5s.

To investigate the change in the ligand concentration upon washing for NCs capped with dynamic and static ligands, we measured the relaxation times of the vinylic protons of bound OGB and OPEA using inversion recovery and compared the evolution of the ligand (by NMR) and particle concentrations (by UV/Vis). As the T_1 of the vinylic protons was determined to be 0.9 and 0.7 s, respectively, and we normalized to the residual signal of the deuterated benzene (T_1 = 8.4 s), we set the recycle delay to 42 s.

^{1}H DOSY and ligand coverage experiments were conducted on an AVANCE III HD (Bruker) spectrometer at 11.7 T (500 MHz) using a 5 mm PABBO probe with 50 G/cm maximum Z-gradient. Diffusion measurements were carried out using a double-stimulated echo sequence with two spoil gradients as proposed by Jerschow and Mueller (1996 & 1997).[9, 10] The basic DOSY experiment consists of three phases. After the initial excitation, a pulsed gradient is used to tag the spins based on their position in the sample. This is followed by a diffusion delay during which molecules can diffuse and change their position. Finally, a refocusing pulsed gradient is used to recover the signal.[11] The signal is attenuated if the physical location of the spins changes during the diffusion delay. This attenuation increases with the strength and length of the gradient pulses. The length of the gradient pulses is kept constant while the amplitude is varied. The diffusion time (Δ = 200 ms) and length of the gradient pulses (δ = 4 ms) were adjusted for the maximal dynamic range, while a full suppression of the signals for colloidal NC samples was not always possible. The diffusion time is limited on the lower end (200 ms) by the longest possible δ tolerated by the probe head (4 ms). On the upper end, the diffusion time is limited by the T_1 relaxation of the signal, leading to reduced signal and therefore longer measurement times. DOSY traces of once-washed DDAB-capped nanocrystals recorded with diffusion times between 200 and 700 ms are shown with their respective fits below (Fig. S8). The diffusion characteristics were then determined by fitting the signal attenuation as a function of the gradient strength using the Stejskal-Tanner equation. Depending on the ligand, we used either the isolated signal of the double bond or the signal of the terminal methyl group(s).

Two scenarios are possible depending on the relation between the ligands' adsorption/desorption rate and the NMR experiment timescale.

1) For ligands with adsorption/desorption rate faster than the experiment timescale, the attenuation of the signal is best described by a single component:

$$\mathrm{I} = \mathrm{e}^{-\mathrm{D}_{measured}\gamma^2\delta^2\mathrm{g}^2\Delta'}$$

With D being the diffusion coefficient, γ being the gyromagnetic ratio and g being the gradient strength. The only fitted parameter in this scenario is the diffusion coefficient, as all other variables are either experimental parameters or physical constants. The resulting diffusion coefficient is a function of the diffusion coefficient of the free ligand (which varies slightly with concentration, Figure S9, and was measured independently at realistic concentrations), the diffusion coefficient of the colloid (which can be estimated from its solvodynamic radius and was measured from colloids with tightly bound ligands OPEA on nanocrystals of the same size and with minimal fraction of free ligands), and the fraction f of bound ligands in solution. As the first two can be obtained from measuring the ligand at concentrations below the critical micelle concentration, and a colloid with a tight binding ligand, we solve for the bound ligand ratio using:

$$f = \frac{D_{free} - D_{measured}}{D_{free} - D_{bound}}$$

2) For ligands with adsorption/desorption rate slower than the experiment timescale, the signal decay is described by two components with the diffusion coefficients of the free ligand and colloid:

$$\mathrm{I} = \sum_{\mathrm{n}=1}^{2} \mathrm{I}_{0,\mathrm{n}} \cdot \mathrm{e}^{-\mathrm{D}_\mathrm{n}\gamma^2\delta^2\mathrm{g}^2\Delta'}$$

In this case, the fraction of bound ligands arises from the ratio of initial intensities. Here, both diffusion coefficients and the ratio of the intensities are fitted. The obtained values are cross-checked with the diffusion coefficients of the free ligand (considering also the concentration dependence) and the size of the colloid.

$$f = \frac{I_{bound}}{I_{bound} + I_{free}}$$

The bound ligand fraction is a function of the ligand and NC concentration and increases when the samples are washed and free ligands are removed.

DOSY NMR does not directly quantify the dynamicity of ligand binding but instead sets a threshold at the experiment timescale, ranking ligand adsorption/desorption as fast (faster than the NMR experiment) and slow (slower than the NMR experiment). Furthermore, DOSY NMR allows ranking ligands within each group by their binding strength, which defines the fraction of bound ligands *f* through the desorption equilibrium constant.

Based on these considerations, we rank the ligands by their dynamic *vs.* static binding behavior and binding strength within each group. For dynamically bound ligands, attenuation of the signal can be fit with a single component. This includes the oleylammonium/oleate pair and OGB. For statically bound ligands, fitting attenuation of the signal requires introducing the slow component corresponding to the statically bound ligands. This includes LAD, DDAB, ASC18, and all phospholipids.

Comparing both dynamically bound ligands, we first note that a fraction of the OAm/OAcH-capped NCs decompose upon each washing step, while OGB-capped particles can be washed up to three times without losing a significant number of NCs. This explains the observation that the diffusion coefficient of the OGB-capped particles decreases upon washing, while OAm/OAcH-capped particles do not exceed a bound ligand fraction *f* of ~0.7. We, therefore, consider the binding of OAm/OAcH to the NCs weaker compared to OGB:

OAm/OAcH << OGB

Comparing the statically bound ligands, we use a fraction of the bound ligands *f* after the standard washing procedure to rank the ligands by their binding strength as follows:

LAD < DDAB < ASC18 << all studied phospholipids

For all ligands with PC- and PEA-headgroups, the fraction of bound ligands *f* is close to 1, resulting in a nearly single-component attenuation but a low diffusion coefficient related to the NC-bound ligands. These ligands are not only static but also exhibit the strongest binding among all surveyed in this study.

5.2. Mass Spectrometry (MS)

MS was conducted at the Molecular and Biomolecular Analysis Service (MoBiAS) of ETH Zurich.

5.3. Transmission Electron Microscopy (TEM)

TEM images were obtained using a JEOL JEM-2200FS microscope operated at 200 kV.

5.4. UV-Vis optical absorption spectroscopy

UV-Vis absorption spectra for colloidal solutions were collected using a Jasco V670 spectrometer in transmission mode.

5.5. Photoluminescence (PL) and absolute PL Quantum Yield (QY) measurements

A Fluorolog iHR 320 Horiba Jobin Yvon spectrofluorometer equipped with a PMT detector was used to acquire steady-state PL spectra from solutions.

The quantum yield (QY) of the samples was measured in Hamamatsu Quantaurus-QY Plus UV-NIR absolute PL spectrometer (C13534-11) equipped with an integrating sphere. The films for QY measurements were prepared by drop-casting 10 µl of the sample mixed with 50 µl of cyclohexane on the square 10mm x 10mm cleaned glass substrates.

5.6. Total X-ray scattering

All samples were measured at the MS-X04SA beamline of the Swiss Light Source (PSI, Switzerland). The OGB/LAD-capped $CsPbBr_3$ NC colloidal suspensions were loaded inside a Ø = 0.7 mm borosilicate glass capillary, and the data collection was performed using a beam energy of 22 keV (wavelength = 0.563730 Å). Together with the scattering patterns of the samples, the signals of the pure solvents (cyclohexane, mesitylene, and toluene) were also collected, to be added as a blank trace to the DSE-based model.

The scattering from the empty capillary and the sample environment were independently collected in the same experimental conditions to be subtracted from the sample signal. Transmission measurements were performed to estimate the sample transmission and correct the data from X-ray absorption.

Total X-ray scattering data was modeled within the Debye Scattering Equation (DSE) analysis as described earlier[12] using an orthorhombic (*Pbnm*) crystal structure, with refined unit cell parameters reported in Table S1 (ranked from the smallest to the largest nominal size). Two independent bond angles, one hinged on the equatorial bromine (Pb-Br_{eq}-Pb, φ_{AB}) and the other one on the axial bromine (Pb-Br_{ax}-Pb, φ_c), are also reported as an outcome of the crystal structure refinement against experimental data. Size and size dispersion have been modeled using prismatic NCs and a bivariate lognormal distribution. 70 % of twinned NCs were used for all the samples, with a single twin boundary ½<110> [001] per NC added to the atomistic model, in order to reproduce the pseudo-tetragonal average crystal structure suggested by the relative intensity of the superstructure peaks. In the case of 5 nm large OGB-capped NCs, the possible effects from poor solvent trace rescaling (due to residual ligands/other impurities) cannot be discarded. In this case, the Cs site occupancy factor was relaxed (about 10% of vacancy) to balance the mismatch of the second peak in the pattern (110 in cubic notation).

6. Computational study of the surface fitness of ligands

To compare the geometric fit of ligand headgroups such as primary ammonium, guanidinium, and quaternary ammonium, small NC models on the DFT level of theory were created. Initial geometry optimization of the 1.8 nm large NC was carried out using a Slater-orbital DZP basis set and the PBE[13] functional as implemented in the ADF code of AMS (v. 2024.102). These calculations also include scalar relativistic effects using the zeroth-order relativistic approximation. After the initial optimization was complete, one Cs atom was replaced by OAm, OGB, or DDAB before the system was relaxed again.

7. Photocatalytic performance of $CsPbBr_3$ NCs capped with various ligands

A 4-mL vial equipped with a stirring bar was charged with benzyl bromide (100 mg, 0.58 mmol, 1.0 equiv.), $CsPbBr_3$ NCs (1.36 mg of $CsPbBr_3$, 0.0023 mmol, 0.4 mole% based on $CsPbBr_3$), 1 mL of the solvent of choice and N, N-diisopropylethylamine (DIPEA, 227 mg, 1.75 mmol, 3.0 equiv.), then sealed with Parafilm. The vial was then placed in the PhotoRedOx TC box, equipped with a 450 nm blue LED (30 W, 250 V), and stirred for 4 hours at 30°C. At the end of the reaction time, 15 μL of 1,4-dioxane and 0.5 mL $CDCl_3$ were added to the crude mixture. 0.2 mL of the resulting solution was diluted to 0.5 mL with $CDCl_3$ for ^{1}H-NMR analysis to determine the conversion values.

8. Supporting Figures

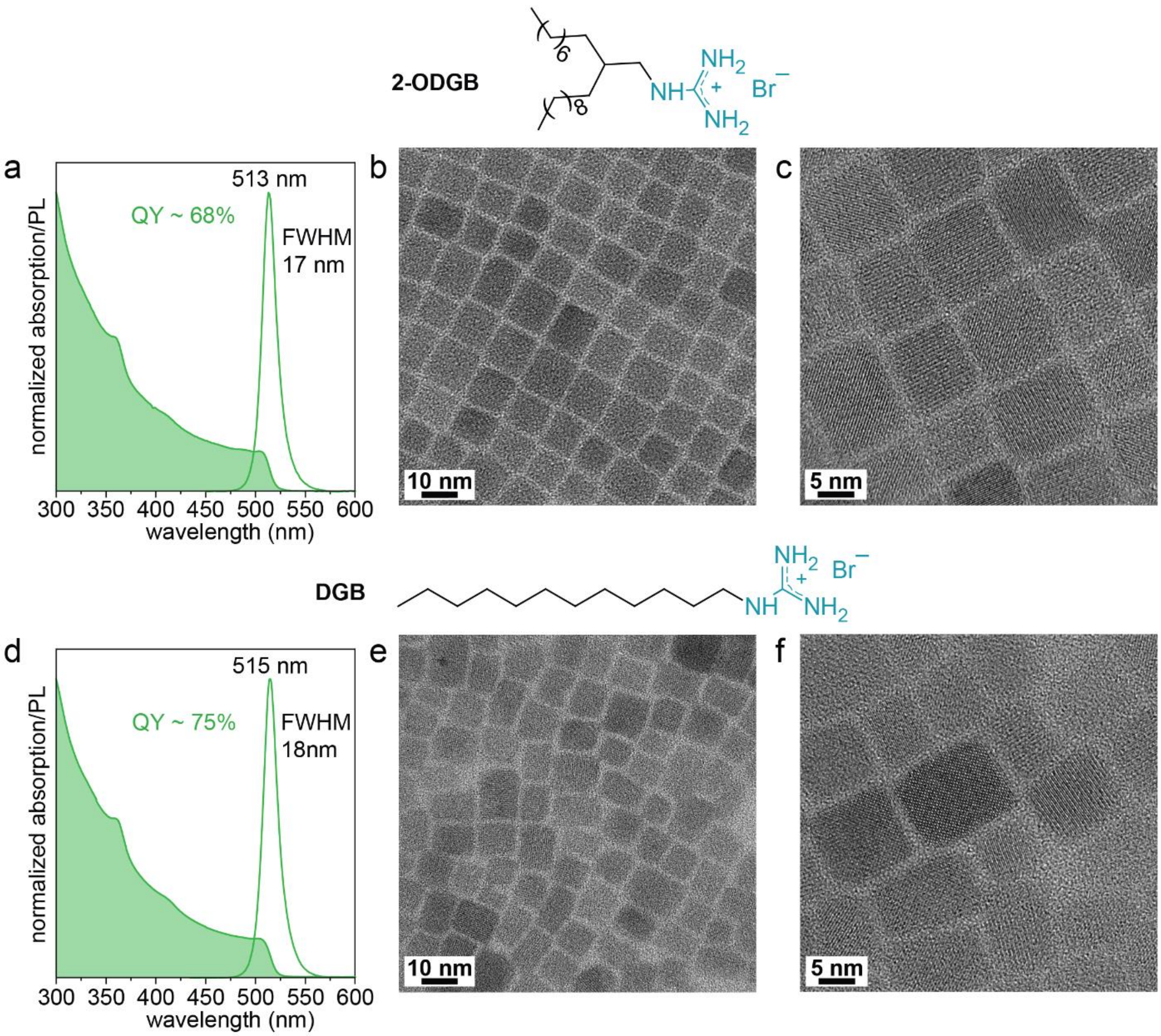

Figure S1. (a) Representative normalized PL and absorption spectra of 2-ODGB-capped $CsPbBr_3$ NCs. (b, c) Corresponding TEM images of 2-ODGB-capped $CsPbBr_3$ NCs. (d) Representative normalized PL and absorption spectra of DGB-capped $CsPbBr_3$ NCs. (e, f) Corresponding TEM images of DGB-capped $CsPbBr_3$ NCs.

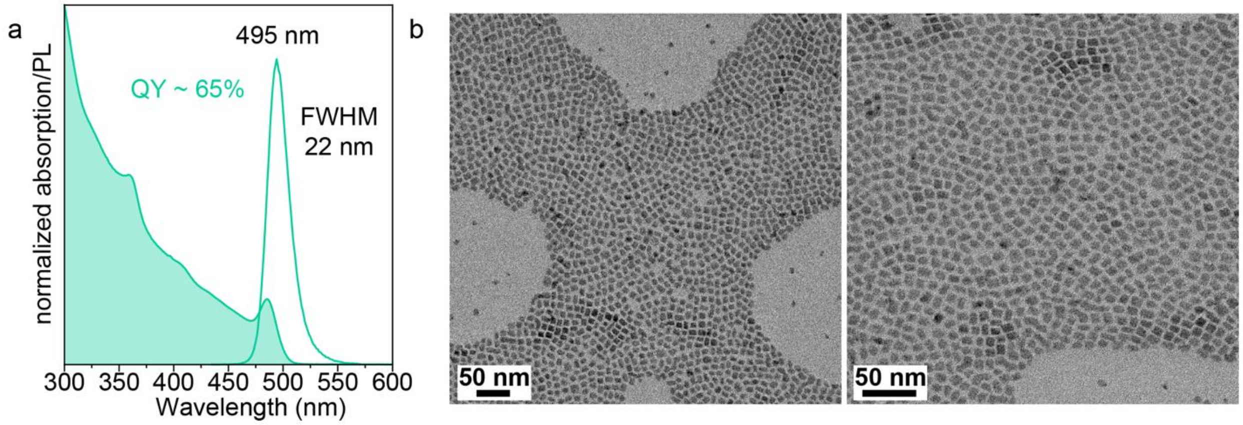

Figure S2. (a) Representative normalized emission and absorption spectra of 5 nm OGB-capped $CsPbBr_3$ NCs. (b) Corresponding STEM images of 5 nm OGB-capped $CsPbBr_3$ NCs.

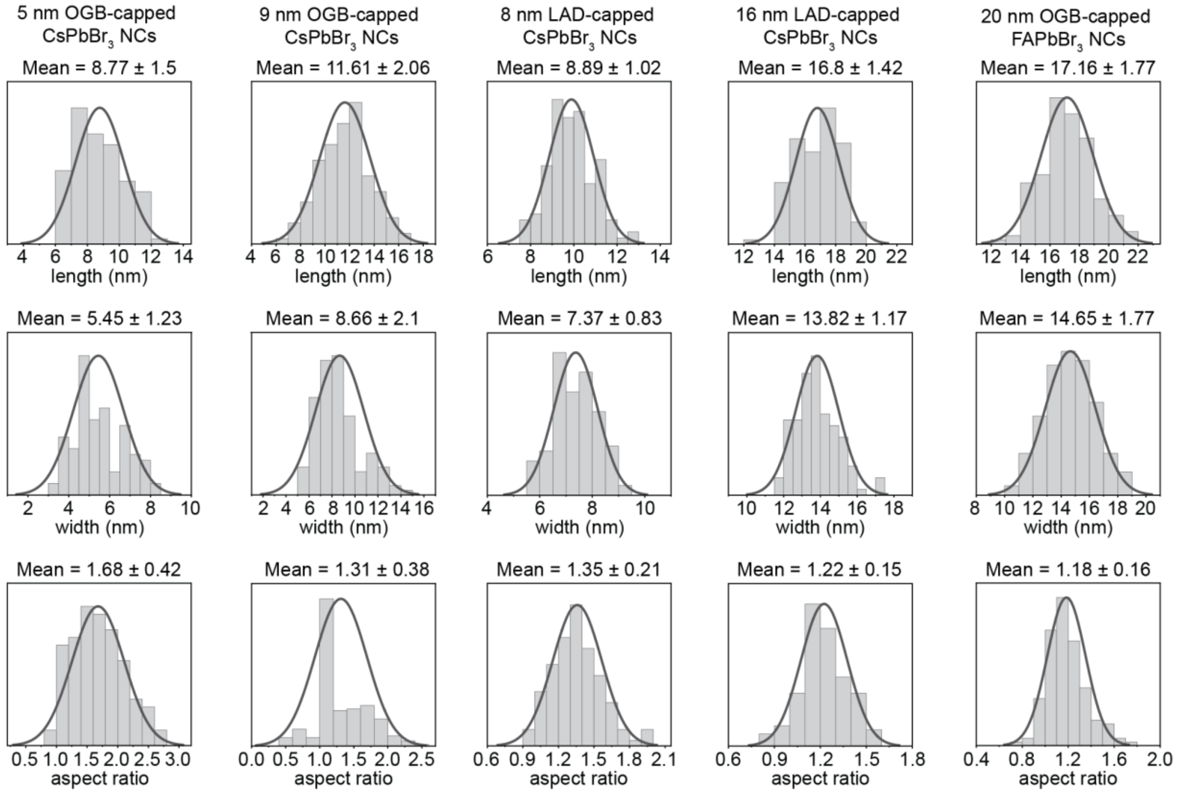

Figure S3. Size and shape distributions of LHP NCs capped with GA-based ligands obtained via hot-injection synthesis. For each sample, histograms of length, width, and aspect ratio (length/width) are shown. Mean values and standard deviations are provided in each panel. Distributions are based on measurements of over 150 NCs per sample from TEM images.

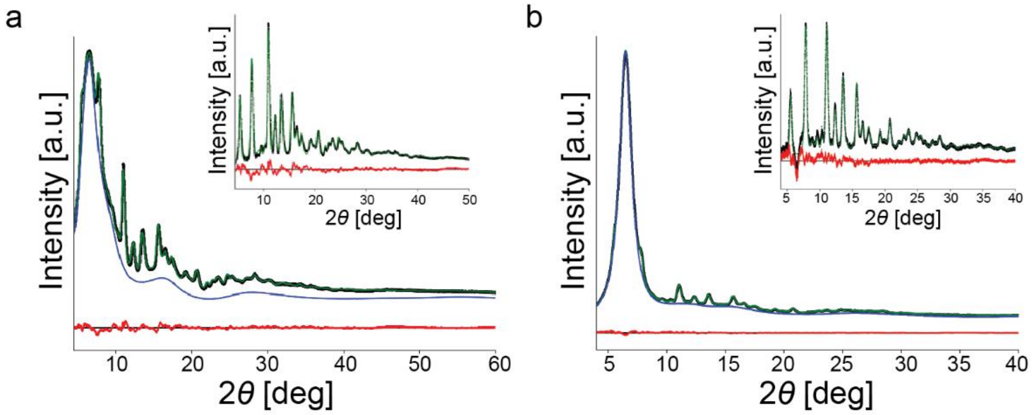

Figure S4. Debye Scattering Equation (DSE) best fit of the synchrotron X-ray total scattering data of OGB-capped $CsPbBr_3$ NCs (wavelength = 0.563730 Å), using the structural model. Experimental data: black dots; calculated pattern: green line; solvent blank trace: blue line; fit residuals: red line. a) 5 nm OGB-capped $CsPbBr_3$ NCs in toluene (GoF = 1.36); b) 9 nm LAD-capped $CsPbBr_3$ NCs in cyclohexane. The insets show the solvent-subtracted traces. Analogous analysis for the 9 nm OGB-capped NCs is reported elsewhere.[12]

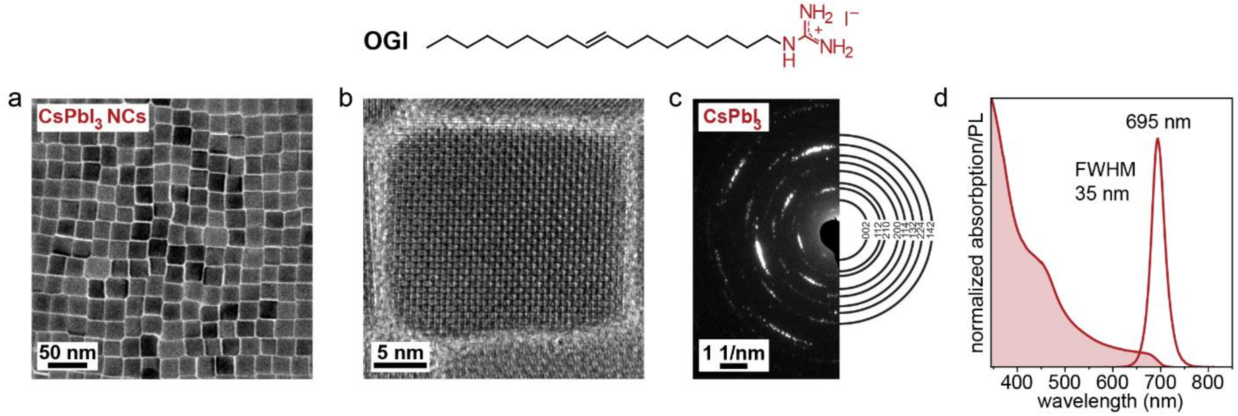

Figure S5. (a, b) TEM and HRTEM images of OGI-capped $CsPbI_3$. (c) Electron diffraction patterns measured from OGI-capped $CsPbI_3$ NCs. (d) Representative normalized emission and absorption spectra.

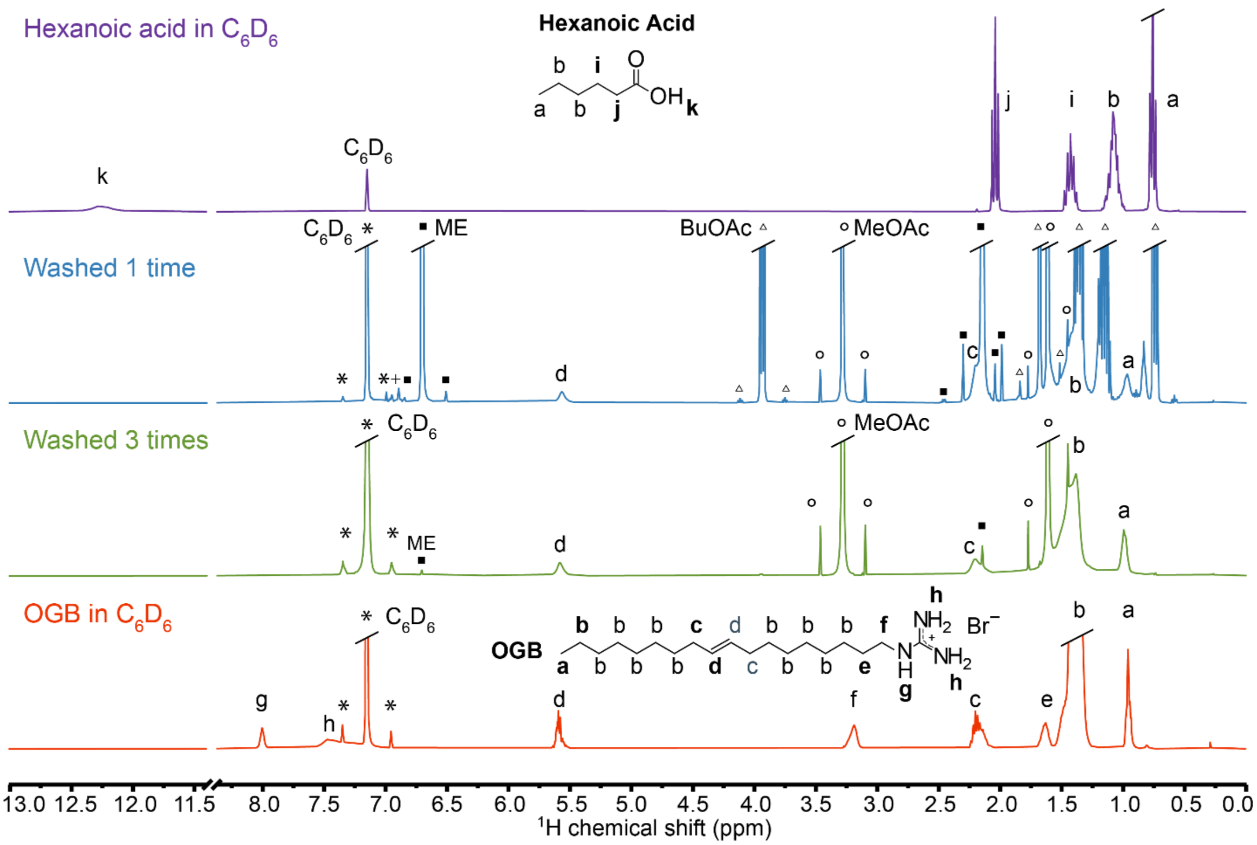

Figure S6. ^{1}H NMR of hexanoic acid (purple), OGB-capped NCs after 1 washing (blue), after 3 washings (green), and OGB itself (orange) in C_6D_6. Peaks common to all solvents (* - benzene-d_6 (C_6D_6), ○ - MeOAc, △ - BuOAc, ■ - Mesitylene (ME)) and components of OGB and hexanoic acid were assigned according to the general pictogram. After the first washing cycle, hexanoic acid is removed to undetectable levels, evidenced by the absence of a peak at ~12.25 ppm in the NMR spectrum.

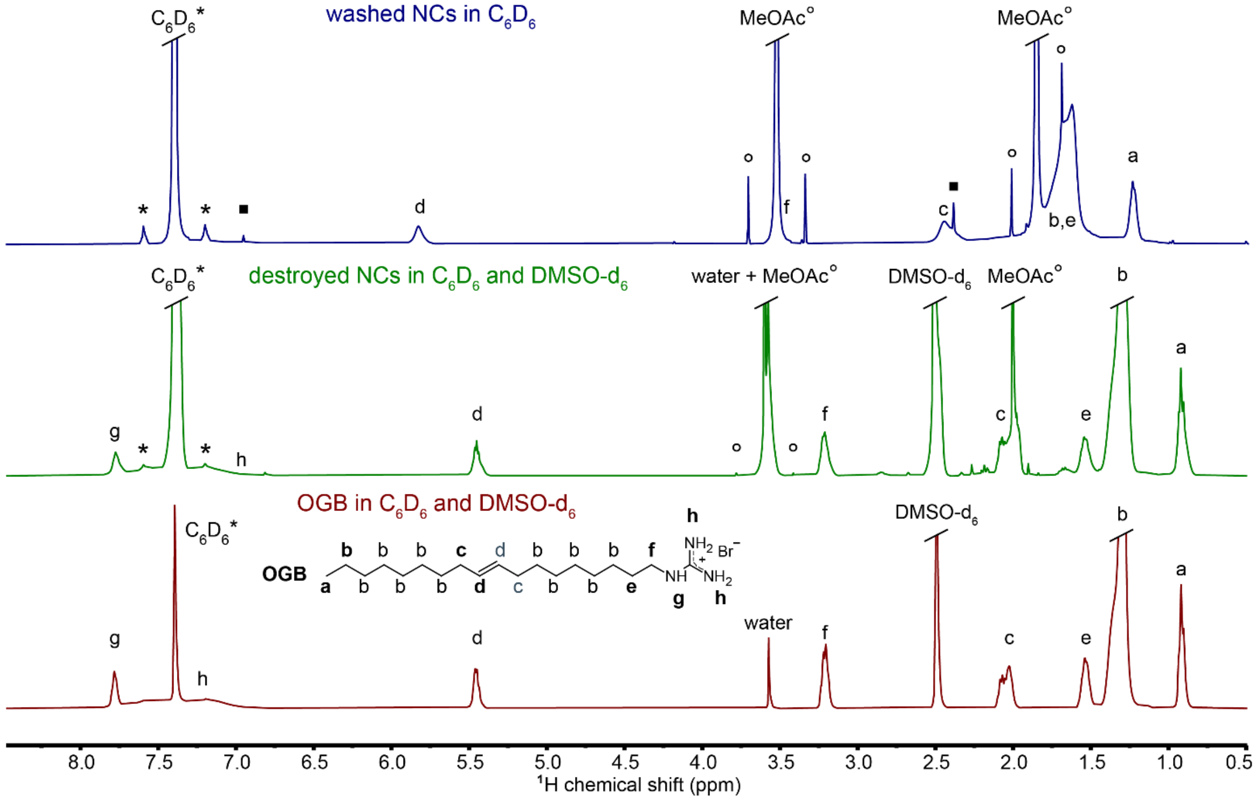

Figure S7. ¹H NMR of OGB-capped NCs after 3 washings (blue) in C_6D_6, decomposed OGB-capped NCs after 3 washings (green), and OGB ligand (red) in a mixture of C_6D_6 and DMSO-d_6. Peaks common to all components of OGB were assigned according to the general pictogram.

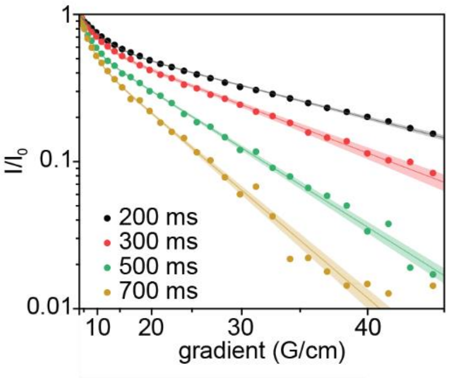

Figure S8. DOSY NMR traces of once-washed DDAB-capped nanocrystal recorded with diffusion times between 200 ms (long enough to allow near full attention of the slowly decaying signal) and 700 ms (the longest diffusion time allowing sufficient signal-to-noise ratio considering the instrument limitations regarding the limited length of the gradient pulses at full power). The lines represent the respective fits, while the shaded area corresponds to the confidence interval of the fit. Neither the individual diffusion coefficients nor the ratio between the two components varies significantly across the measurements, resulting in excellent fits and robust results: D(fast) = 3.5(3) * 10^{-10} m^2/s; D(slow) = 3.15(4) * 10^{-11} m^2/s; f = 0.388(4) (see individual fits below).

200 ms: D(fast) = 3.88 * 10^{-10} m^2/s; D(slow) = 3.20 * 10^{-11} m^2/s; f = 0.37.

300 ms: D(fast) = 3.40 * 10^{-10} m^2/s; D(slow) = 3.15 * 10^{-11} m^2/s; f = 0.36.

500 ms: D(fast) = 3.23 * 10^{-10} m^2/s; D(slow) = 3.11 * 10^{-11} m^2/s; f = 0.38.

700 ms: D(fast) = 3.30 * 10^{-10} m^2/s; D(slow) = 3.14 * 10^{-11} m^2/s; f = 0.44.

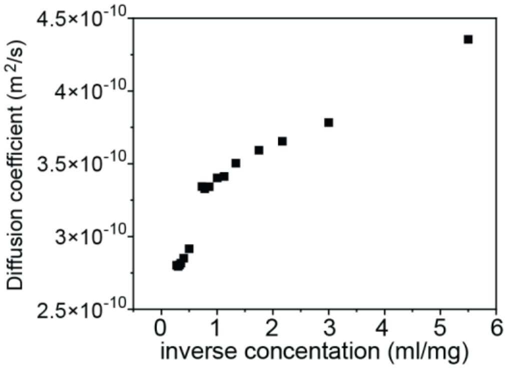

Figure S9. Concentration-dependent diffusion coefficient of free DDAB ligand in benzene.

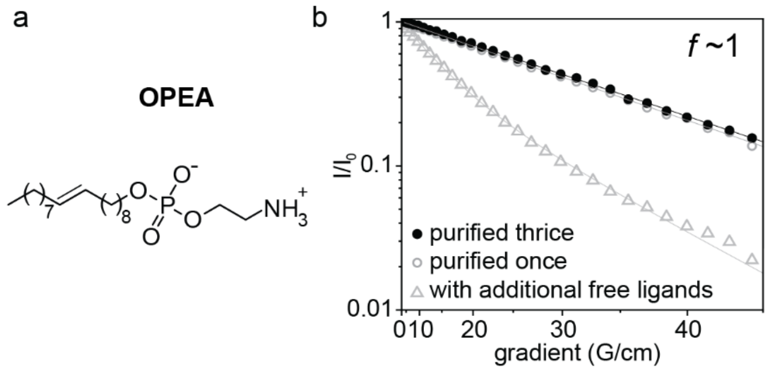

of ligand.

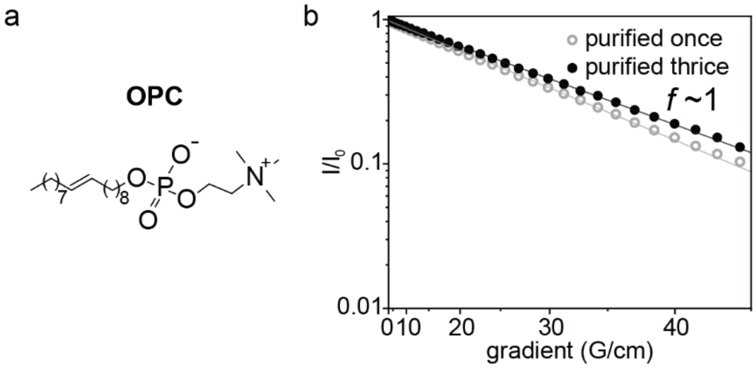

Figure S11. (a) Chemical structure of OPC. (b) Measured DOSY NMR traces of once and thrice washed OPC-capped $CsPbBr_3$ NCs. The lines represent the respective fits.

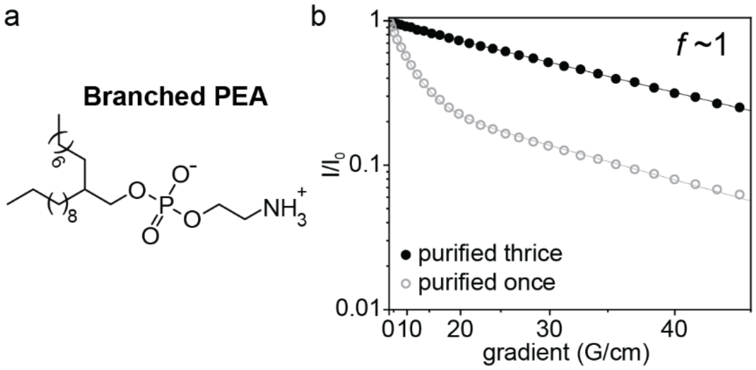

Figure S12. (a) Chemical structure of branched PEA (brPEA). (b) Measured DOSY NMR traces of once and thrice washed brPEA-capped $CsPbBr_3$ NCs. The lines represent the respective fits.

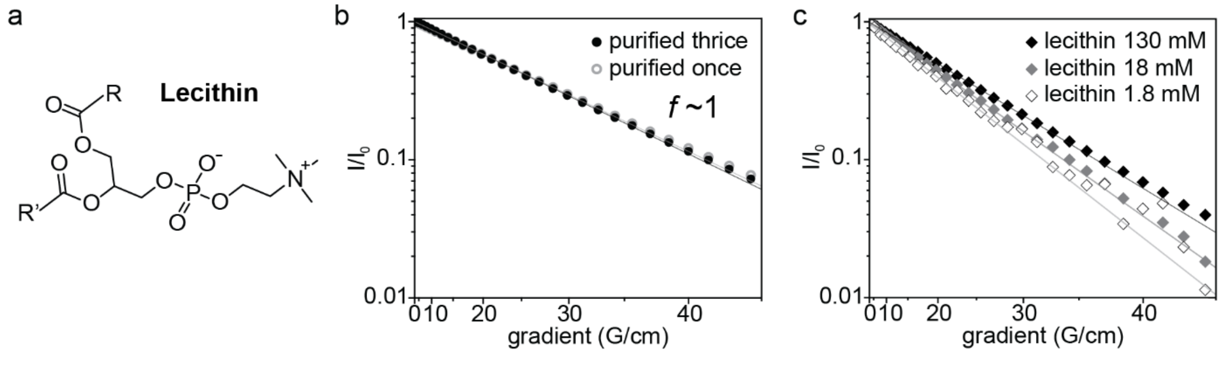

Figure S13. (a) Chemical structure of lecithin, where R, R’ = fatty acids residue. (b) Measured DOSY NMR traces of once and thrice washed lecithin-capped $CsPbBr_3$ NCs. The lines represent the respective fits. (c) Measured DOSY NMR traces of lecithin at different concentrations.

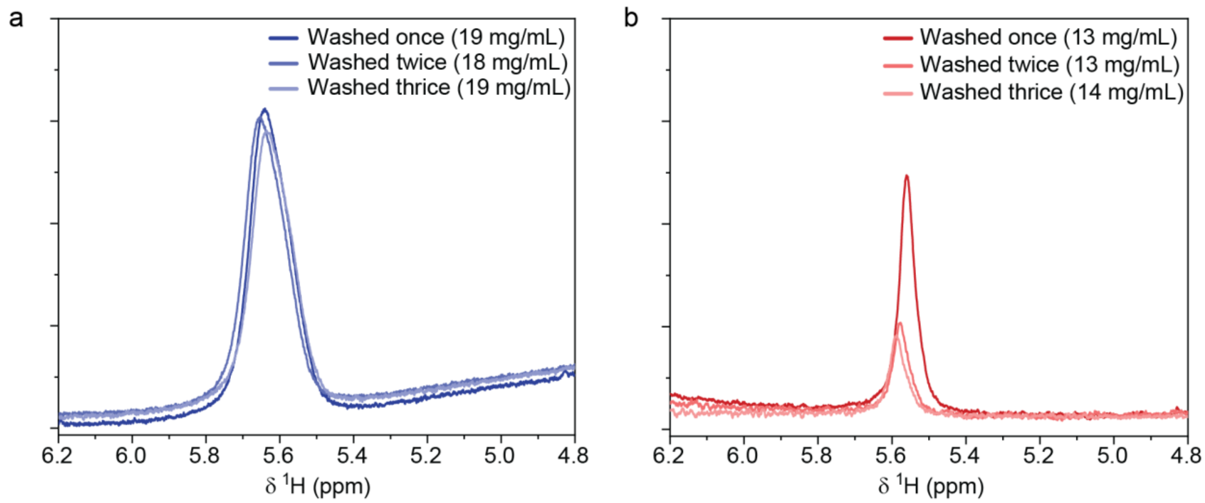

Figure S14. (a) ^{1}H NMR spectra of OPEA-capped $CsPbBr_3$ NCs after one, two, and three purification cycles. The spectra were scaled based on the integral of the benzene solvent peak. (b) Proton spectra of OGB-capped $CsPbBr_3$ NCs after one, two and three washing cycles. The spectra were scaled based on the integral of the benzene solvent peak. From the obtained spectra, we can clearly identify that while the ligand concentration for the static ligand remains nearly unchanged, the ligand concentration of the OGB-capped particles is reduced with each washing step, coinciding with the observed change in diffusion coefficient.

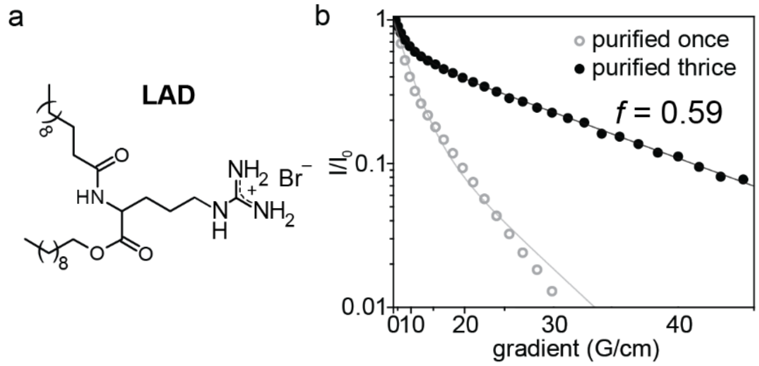

Figure S15. (a) Chemical structure of LAD. (b) Measured DOSY NMR traces of once and thrice washed LAD-capped $CsPbBr_3$ NCs. The lines represent the respective fits.

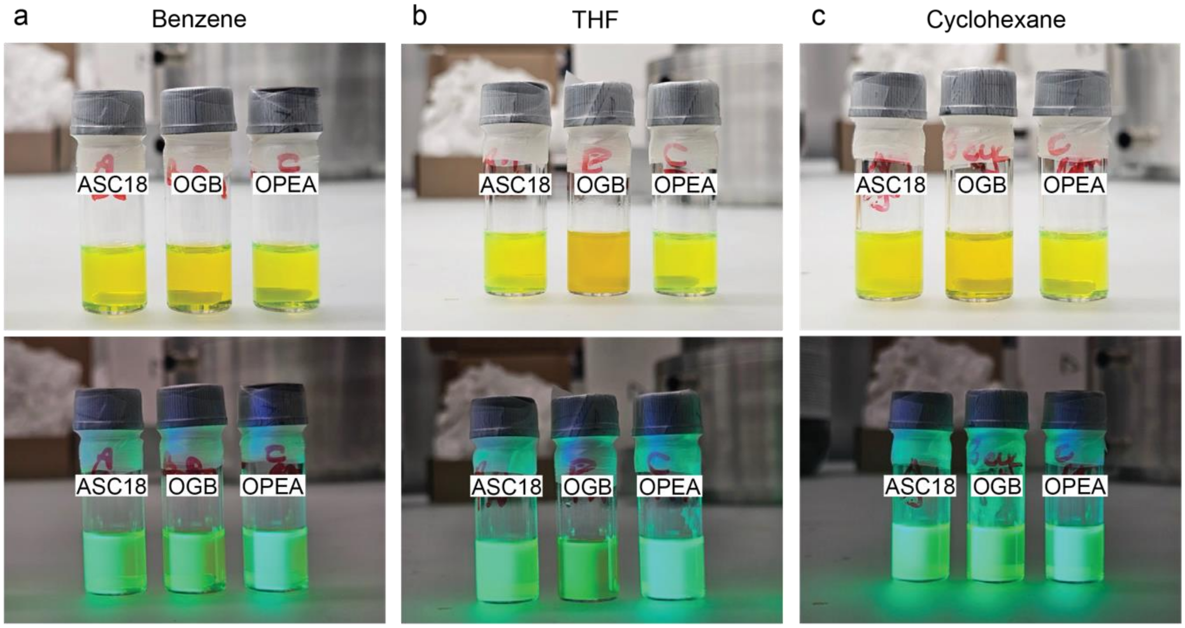

Figure S16. The photographs of the reaction mixture of substrate and colloidal solution of $CsPbBr_3$ NCs capped with ASC18/OGB/OPEA before the catalytic reaction under normal light (above) and UV light (below), dissolved in (a) Benzene, (b) THF, and (c) Cyclohexane.

9. Supporting Tables

Table S1. Refined unit cell parameters and volume obtained from X-Ray analysis according to the orthorhombic Pbnm crystal symmetry. Refined φ_{AB} and φ_c are the bond angles hinged on the equatorial and axial bromine, respectively, which can be used as proxies of octahedra tilts.

sample	size from TEM (nm)	a(Å)	b(Å)	c(Å)	V (Å^3)	φ_{AB}(°)	φ_c(°)
OGB-capped $CsPbBr_3$ NCs	5.5	8.176(1)	8.346(1)	11.8290(6)	807.2(1)	159.31(5)	167.1(2)
	9	8.1982(7)	8.3196(6)	11.8069(9)	805.3(1)	157.10(4)	165.1(2)
LAD-caped $CsPbBr_3$ NCs	8	8.184(1)	8.3028(9)	11.797(1)	801.6(2)	157.1(1)	163.8(2)

Table S2. Results of DSE-based size analysis of guanidinium-capped $CsPbBr_3$ NCs experimental data: $\langle L_a \rangle$ and $\langle L_c \rangle$ the average prism edges, projected along the two crystallographic directions (assuming a prismatic NCs morphology, with $\langle L_a \rangle \sim \langle L_b \rangle$) and $\sigma/\langle L_x \rangle$ the relative standard deviations of the number-based lognormal size distribution function used to model the NCs size dispersion. $\langle L_K \rangle$ is the average edge of nanocubes of equivalent volume. Analysis shows that guanidinium-capped $CsPbBr_3$ NCs have a cuboidal, slightly prolate along the c-axis, shape with an aspect ratio of 1.2-1.3 (1.6 for small size), in agreement with TEM analysis. DSE analysis reveals a larger polydispersity along the c-axis (22 and 24 % for OGB- and LAD-capped NCs, respectively), while the lateral size distribution is very uniform (6 and 14 %) for the kinetic growth conditions.

sample	size from TEM (nm)	$\langle L_a \rangle$ (nm), $\sigma/\langle L_{ab} \rangle$	$\langle L_c \rangle$ (nm), $\sigma/\langle L_c \rangle$	aspect ratio $\langle L_c \rangle/\langle L_a \rangle$	$\langle L_K \rangle$ (nm), $\sigma/\langle L_{eq} \rangle$
OGB-capped $CsPbBr_3$ NCs	5.5	4.64, 0.09	7.50, 0.16	1.61	5.45, 0.08
	9	6.96, 0.06	9.14, 0.22	1.32	7.62, 0.09
LAD-caped $CsPbBr_3$ NCs	8	6.36, 0.14	7.52, 0.24	1.2	10.45, 0.09

Table S3. Fitting results of the DOSY NMR traces.

Sample	D_{fast} (m^2/s)	D_{slow} (m^2/s)	f
Free OAm	6.20(1) *10^{-10}	-	-
OAm-capped NCs, washed once	2.51(2) *10^{-10}	-	0.64
OAm-capped NCs, washed twice	2.2(2) *10^{-10}	-	0.68
Free OGB	1.448(6) *10^{-10}	-	-
OGB-capped NCs, washed once	1.42(2) *10^{-10}	-	0.02

OGB-capped NCs, washed thrice	$4.98(3) *10^{-11}$	-	0.97
Free DDAB	$2.92(4) *10^{-10}$	-	-
DDAB-capped NCs, washed once	$3.68(1) *10^{-10}$	$3.23(5) *10^{-11}$	0.378(6)
DDAB-capped NCs, washed twice	$4.27(1) *10^{-10}$	$6.0(2) *10^{-11}$	0.671(1)
Free ASC18	$3.3(2) *10^{-10}$	-	-
ASC18-capped NCs, washed once	$7.26(9) *10^{-10}$	$5.25(1) *10^{-11}$	0.280(4)
ASC18-capped NCs, washed thrice	$1.10(1) *10^{-9}$	$4.65(4) *10^{-11}$	0.883(5)
Free OPEA	$1.54(5) *10^{-10}$	-	-
OPEA-capped NCs, washed once	-	$4.46(2) *10^{-11}$	~1
OPEA-capped NCs, washed thrice	-	$4.33(2) *10^{-11}$	~1
OPEA-capped NCs, with excess ligand	$3.77(3) *10^{-10}$	$6.27(1) *10^{-10}$	0.719(2)
brPEA-capped NCs, washed once	$3.7(6) *10^{-10}$	$3.10(2) *10^{-11}$	0.28
brPEA-capped NCs, washed thrice	$4.05(4) *10^{-10}$	$3.55(7) *10^{-11}$	0.95
OPC-capped NCs, washed once	-	$5.36(4) *10^{-11}$	~1
OPC-capped NCs, washed thrice	-	$4.72(2) *10^{-11}$	~1
Free LAD (high conc.)	$1.68(1) *10^{-10}$	-	-
LAD-capped NCs, washed once	$4.8(6) *10^{-10}$	$1.2(5) *10^{-10}$	0.2(1)
LAD-capped NCs, washed thrice	$5.0(4) *10^{-10}$	$4.75(2) *10^{-11}$	0.58(2)
Free Lecithin (low conc.)	$9.15(5) *10^{-11}$	-	-
Free Lecithin	$8.68(8) *10^{-11}$	-	-
Free Lecithin (high conc.)	$7.85(3) *10^{-11}$	-	-
Lecithin-capped NCs, washed once	-	$6.12(2) *10^{-11}$	~1
Lecithin-capped NCs, washed thrice	-	$6.26(3) *10^{-11}$	~1

10. Supporting information references

(1) Protesescu, L.; Yakunin, S.; Bodnarchuk, M. I.; Krieg, F.; Caputo, R.; Hendon, C. H.; Yang, R. X.; Walsh, A.; Kovalenko, M. V. Nanocrystals of Cesium Lead Halide Perovskites ($CsPbX_3$, X = Cl, Br, and I): Novel Optoelectronic Materials Showing Bright Emission with Wide Color Gamut. *Nano Lett.* **2015**, *15* (6), 3692-3696. DOI: 10.1021/nl5048779.

(2) Shynkarenko, Y.; Bodnarchuk, M. I.; Bernasconi, C.; Berezovska, Y.; Verteletskyi, V.; Ochsenbein, S. T.; Kovalenko, M. V. Direct Synthesis of Quaternary Alkylammonium-Capped Perovskite Nanocrystals for Efficient Blue and Green Light-Emitting Diodes. *ACS Energy Lett.* **2019**, *4* (11), 2703-2711. DOI: 10.1021/acsenergylett.9b01915.

(3) Krieg, F.; Ochsenbein, S. T.; Yakunin, S.; ten Brinck, S.; Aellen, P.; Suess, A.; Clerc, B.; Guggisberg, D.; Nazarenko, O.; Shynkarenko, Y.; et al. Colloidal $CsPbX_3$ (X = CI, Br, I) Nanocrystals 2.0: Zwitterionic Capping Ligands for Improved Durability and Stability. *ACS Energy Lett.* **2018**, *3* (3), 641-646. DOI: 10.1021/acsenergylett.8b00035.

(4) Akkerman, Q. A.; Nguyen, T. P. T.; Boehme, S. C.; Montanarella, F.; Dirin, D. N.; Wechsler, P.; Beiglböck, F.; Rainò, G.; Erni, R.; Katan, C.; et al. Controlling the nucleation and growth kinetics of lead halide perovskite quantum dots. *Science* **2022**, *377* (6613), 1406-1412. DOI: 10.1126/science.abq3616.

(5) Morad, V.; Stelmakh, A.; Svyrydenko, M.; Feld, L. G.; Boehme, S. C.; Aebli, M.; Affolter, J.; Kaul, C. J.; Schrenker, N. J.; Bals, S.; et al. Designer phospholipid capping ligands for soft metal halide nanocrystals. *Nature* **2024**, *626* (7999). DOI: 10.1038/s41586-023-06932-6.

(6) Aoyagi, N.; Furusho, Y.; Endo, T. Convenient Synthesis of Acyclic Guanidines from Isothiouronium Iodides and Amines without Protection of the Amino Groups. *Synlett* **2014**, *25* (07), 983-986. DOI: 10.1055/s-0033-1340904.

(7) Yamato, N.; Yumioka, R. Cosmetic composition. US2003165448A1, 2003.

(8) Ghorbani-Choghamarani, A.; Taherinia, Z. Synthesis of peptide nanofibers decorated with palladium nanoparticles and its application as an efficient catalyst for the synthesis of sulfides via reaction of aryl halides with thiourea or 2-mercaptobenzothiazole. *RSC Advances* **2016**, *6* (64), 59410-59421, 10.1039/C6RA02264B. DOI: 10.1039/C6RA02264B.

(9) Jerschow, A.; Müller, N. 3D Diffusion-Ordered TOCSY for Slowly Diffusing Molecules. *Journal of Magnetic Resonance, Series A* **1996**, *123* (2), 222-225. DOI: 10.1006/jmra.1996.0241.

(10) Jerschow, A.; Müller, N. Suppression of convection artifacts in stimulated-echo diffusion experiments. Double-stimulated-echo experiments. Academic Press: 1997; Vol. 125, pp 372-375.

(11) Morris, G. A. Diffusion-ordered spectroscopy. *eMagRes* **2007**.

(12) Bertolotti, F.; Dengo, N.; Cervellino, A.; Bodnarchuk, M. I.; Bernasconi, C.; Cherniukh, I.; Berezovska, Y.; Boehme, S. C.; Kovalenko, M. V.; Masciocchi, N.; et al. Size- and Temperature-Dependent Lattice Anisotropy and Structural Distortion in $CsPbBr_3$ Quantum Dots by Reciprocal Space X-ray Total Scattering Analysis. *Small Struct.* **2024**, *5* (3), 2300264. DOI: 10.1002/sstr.202300264.

(13) Perdew, J. P.; Burke, K.; Ernzerhof, M. Generalized Gradient Approximation Made Simple. *Phys. Rev. Lett.* **1996**, *77* (18), 3865-3868. DOI: 10.1103/PhysRevLett.77.3865.
